# Do Contemplative Practices Promote Trauma Recovery? A Narrative Review from 2018 to 2023

**DOI:** 10.3390/healthcare13222825

**Published:** 2025-11-07

**Authors:** Francesca Scafuto, Rossella Mattea Quinto, Graziella Orrù, Alessandro Lazzarelli, Rebecca Ciacchini, Ciro Conversano

**Affiliations:** 1 Department of Languages and Literatures, Communication, Education and Society, University of Udine, 33100 Udine, Italy; 2 Department of Health and Life Sciences, European University of Rome, 00163 Rome, Italy; rossellamattea.quinto@unier.it; 3 Department of Surgical, Medical and Molecular Pathology and Critical Care Medicine, University of Pisa, 56126 Pisa, Italy; graziella.orru@unipi.it (G.O.); ciro.conversano@unipi.it (C.C.); 4 Department of Civilizations and Forms of Knowledge, University of Pisa, 56126 Pisa, Italy; a.lazzarelli@gmail.com; 5 School of Advanced Studies, University of Camerino, 62032 Camerino, Italy; rebecca.ciacchini@med.unipi.it

**Keywords:** contemplative practices, trauma, trauma recovery, trauma-related symptoms, mindfulness, yoga, narrative review

## Abstract

**Background:** Contemplative practices encompass a variety of static and dynamic practices. These practices, by fostering insights, heightened awareness, and a deeper connection to a broader framework of meaning, may play a crucial role in significantly reducing trauma-related symptoms in both young and adult populations. **Methods:** The current narrative literature review used Scopus and PubMed to search for studies published between January 2018 and August 2023 that examined the effects of contemplative practices, an umbrella term that includes mindfulness-based interventions, yoga, tai chi, qigong, and meditation, on trauma recovery and PTSD symptoms among adults and youths. **Results:** The literature search identified 281 articles. Forty-two studies met the inclusion criteria and were critically evaluated. Among the various approaches, encompassing stand-alone contemplative practices and combined interventions, mindfulness emerged as the most employed and investigated practice for supporting trauma recovery. Contemplative practices have been shown to effectively reduce various dimensions of traumatic experience, such as reactivity, intrusion, hyperarousal, and negative cognitions and mood. Controversial results were found on avoidance symptoms and physiological parameters. **Conclusions:** The results give support to the idea of combining contemplative practices with trauma-focused psychotherapeutic interventions to foster a sense of safety and enhance emotional expression and awareness of feelings of fear, shame, guilt, or inferiority while improving metacognitive processes. This, in turn, supports healing the sense of self, restoring a sense of basic trust in self and others, which is often deeply affected in individuals who have experienced trauma.

## 1. Introduction

### 1.1. Contemplative Practices: Theoretical Frameworks

Contemplative practices appear to be an umbrella term, including a variety of static and dynamic practices, such as mindfulness meditation, but also embodied and movement-based practices, such as tai chi, qigong, and yoga. They often require formal activities for 20 min or more, once or twice daily [1,2]. Religious practices (e.g., prayer and chanting) also can be included in the range of contemplative practices. Despite most studies considering contemplative practices a broader term that also includes mindfulness meditation, some scholars underline crucial differences.

Meditative practices, as described by Sparby and Sacchet [3], are widely used today for their health benefits, emphasizing direct and non-judgmental experience rather than analytical reflection, and they are often detached from their original spiritual aims. In contrast, contemplative practices are directed toward specific spiritual goals such as *liberation*, *enlightenment*, or *awakening* [4]. In the Christian contemplative tradition, *contemplatio*—from the Latin *contemplari* (cum templum)—denotes an effortless state of joy and intimate union with the divine, as described by Thomas Aquinas and Teresa of Ávila [5]. While the meditative life cultivates insight into truth, the contemplative life deepens the love of truth, pacifying cognition, memory, and will to awaken the divine nature within. Similarly, in the Dzogchen tradition, Tibetan masters such as Chogyal Namkhai Norbu define contemplation as the culmination of meditation: a non-dual, self-liberating state free from mental effort, in which the distinction between knower and known dissolves [6,7]. Among the various contemplative forms, mindfulness meditation, encompassing attentional (e.g., focused attention, open monitoring) and constructive practices, remains the most widespread in the West [8].

Within the context of trauma recovery, constructive contemplative practices play an important role because they specifically aim to cultivate positive emotional states that counterbalance the effects of stress and trauma. Among these, loving-kindness, compassion, and self-compassion practices are particularly relevant [9]. Loving-kindness meditation (LKM) involves intentionally generating warm, caring, and positive feelings toward oneself and others, often through silently repeating phrases or visualizing the extension of goodwill to different people [10]. Compassion meditation, on the other hand, emphasizes empathy and concern for those who suffer, with the goal of strengthening prosocial motivation and supportive behavior. The broaden-and-build theory helps explain the relevance of these practices: while negative emotions arise from perceived threats and trigger defensive responses such as fight-or-flight, positive emotions expand awareness, foster openness to new experiences, and build long-term psychological resources [11].

The growing body of research on contemplative practices testifies the peak attention of researchers over recent decades, focusing on the melting point between Eastern and Western approaches to science and recognizing psychosocial and psychobiological benefits in clinical and non-clinical settings [1]. Originally, these practices were designed to investigate the essence of reality in pursuit of truth, rather than as methods. However, many studies have revealed the therapeutic effects in several psychological domains with a decrease in symptoms, emotional and behavioral problems [12,13], and an increase in well-being outcomes [14], and real happiness [15].

### 1.2. Trauma and Post-Traumatic Symptoms: Recovery Pathways Through Contemplative Practices

The association between trauma-related symptoms, emotion dysregulation, and contemplative practices has been investigated in several previous reviews and meta-analyses [2,11,16,17]. According to the DSM-5, post-traumatic stress disorder (PTSD) is defined by a qualifying exposure to a traumatic event that includes “actual or threatened death, serious injury, or sexual violence” [18]. There are four main types of qualifying exposure: direct personal exposure, witnessing of trauma to others, indirect exposure through trauma experience of a family member or other close associates, and repeated or extreme exposure to aversive details of a traumatic event [19]. The DSM-5 lists 20 symptoms along four groups: intrusion (unwanted and involuntary re-experiencing of the traumatic memories in a person’s daily life), avoidance (steering clear of people, places, feelings, or thoughts that remind of the trauma), negative alterations in cognition and mood (e.g., negative self-perception, blame, guilty), and alterations in arousal and reactivity (e.g., heightened alertness, difficulties in managing emotional and physical responses, concentration problems, sleep disturbances).

Trauma recovery refers to the multifaceted process through which individuals heal from the psychological, emotional, and physiological impacts of traumatic experiences. This process often involves restoring a sense of safety, re-establishing trust in oneself and others, and reintegrating fragmented aspects of identity and functioning. Judith Herman [20] delineates trauma recovery into three stages: establishing safety, retelling the traumatic story, and reconnecting with others. These stages emphasize the importance of creating a safe environment, processing traumatic memories, and rebuilding relationships as central components of the healing process.

In this context, mindfulness-based interventions for PTSD have demonstrated clinically meaningful improvement in PTSD symptoms immediately post-treatment [21]. Embodied contemplative practices are supposed to work better for trauma [22] as well as somatic therapies, in comparison with conventional psychotherapies. Indeed, considering polyvagal theory and the stress-reactive dissociation of traumatic experience from prefrontal modulation and left-sided verbal processing, discursive psychotherapy is less likely to work on trauma than somatic approaches, including psychodramatic re-enactments, narrative and poetic reframing, posture yoga, and intensive breath-work [23].

Embodied contemplative practices consist of two broad types of methods: top-down (i.e., the downregulation starting from mental processing at the level of the cerebral cortex) and bottom-up (i.e., pathways from the periphery to the brainstem and cerebral cortex) [24]. Both work supporting implicit memory and procedural conditioning to calm the post-traumatic reactivity [25]. Top-down embodied practices include, for instance, corrective body imagery (visualization), prosocial prosody (recitation); bottom-up embodied practices include, for instance, expressive movement (posture–gesture) and energizing breath-activation (e.g., kundalini yoga). Movement-based contemplative practices, such as tai chi, qigong, and yoga, can be seen as bottom-up practices that emphasize the use of spontaneous and active movement and breathing, playing a key role in the regulation of both the autonomic nervous system and hypothalamic–pituitary–adrenal axis [26], which are central to PTSD and stress physiological symptoms [27]. Furthermore, movement-based contemplative practices may reduce PTSD symptoms by increasing interoceptive ability [23,28], which is crucial in emotional experience and regulation [29,30].

Contemplative practices, characterized by the attentive regulation of breathing or of body movements and postures, are believed to affect vagal activation. VVC is activated, for instance, by glossopharygeal and auditory feedbacks that derive from expressing soothing vocal tones in prayer or chanting [31,32]. Imagining or voluntary rhythmic breathing activates the smart vagus and its medullary nucleus, regulating reflex autonomic stress-reactivity [33]. Similarly, rhythmic movement and deep abdominal breathing activate feedbacks sent to the solitary nucleus from visceral vagal afferents, promoting the bottom-up regulation of stress reactivity [34].

According to polyvagal theory [32,35], contemplative practices affect trauma responses since they can be seen as neural exercises expanding the capacity of the ventral vagal complex to regulate the present state and to increase neuroception of safety and social engagement system rather than threat. Indeed, contemplative practices were found to increase baseline Respiratory Sinus Arrhythmia (RSA), which reflects the activation of the ventral vagal complex (VVC), thus promoting PTSD recovery [2].

Finally, an interesting perspective is given by emancipatory contemplative practices, which are aimed at intentionally creating opportunities to access our embodied cultural, collective, and ancestral memory. The focus of these practices is the decolonization of traditional wisdom of indigenous people that has been stolen or erased, starting from the reconnection to our bodies, hearts, and minds [36]. Trauma is framed in an intergenerational, historical, and collective context, hence the healing process is culturally grounded and based on connecting to inner aliveness through soulful experiencing, coming into harmony with nature and in alignment with the activity of spirit [37]. An example is the contemplative practice called *Rising Up Rooted*, offered as a methodology for awakening Black wisdom [38]. Indeed, Black cultural expressions (e.g., music, dance, poetry, spoken word) and cultural practices (e.g., rituals, ceremonies) can be powerful means for cultural connection and healing trauma [39,40].

While the theoretical rationale for the benefits of contemplative practices in trauma recovery is strong, more empirical evidence is still needed to address methodological limitations, such as small sample sizes and lack of randomized control groups in clinical trials [41]. Furthermore, one of the prominent issues that makes the investigation still needed is the report of several side-effects, most commonly revealed as an increase in anxiety, traumatic re-experiencing, and emotional sensitivity [42].

Some side-effects of contemplative practices may be understood as part of the meditative process, as discomfort may accompany both healing and meditative insights [43]. Early Buddhist accounts also acknowledge such challenges along the meditative path (e.g., [44]). However, when initial discomfort is overcome and traumatic experiences and symptoms repertory are elaborated, trauma can become an opportunity of personal growth. Indeed, participants reporting MRAE were as equally glad to have practiced meditation as those not reporting MRAE [42]. Reflecting in depth about a traumatic event tends to promote a search for new purpose and meaning in one’s life, a process that has been documented as post-traumatic growth [45].

### 1.3. Aims

The aim of this narrative review was to evaluate the role of contemplative practices in trauma recovery, considering both stand-alone interventions and those integrated within established psychotherapeutic approaches. Specifically, we sought to examine how different types of interventions, ranging from pure contemplative practices, such as mindfulness meditation, loving-kindness meditation, and yoga, to psychotherapeutic programs that incorporate contemplative elements like CBT or ACT, affect trauma-related outcomes. In achieving this, we aimed to capture key intervention characteristics, including type, duration, frequency, and delivery format, as well as the primary outcome domains assessed, such as symptom reduction (e.g., PTSD, anxiety, depression), emotion regulation, attentional control, psychological and social well-being, dispositional mindfulness, psychophysiological parameters, and overall functioning. By systematically considering both intervention features and outcome variables, we intended to provide a comprehensive understanding of the potential contribution of contemplative practices to trauma recovery.

## 2. Methods

### 2.1. Search Strategy

The present narrative review was conducted to examine the effects of contemplative practices on trauma recovery. Two databases were selected for this review: Scopus and Pubmed. The search was performed on 7 August 2023, restricting the search to studies published from 1 January 2018, in light of an existing review (i.e., [46]). Finally, the references of the included papers were also examined to potentially identify additional articles not found in the search.

The search strategy included the following search terms: (“Historical trauma” OR “Sexual trauma” OR “Psychological Trauma” OR “Trauma and Stressor Related Disorders” OR “Stress Disorders, Traumatic, Acute” OR “Stress Disorders, Post-Traumatic”) AND (“Contemplative practices” OR “Mindfulness” OR “Meditation” OR “Yoga” OR “Tai chi” OR “Tai Ji” OR “Qigong” OR “Self-compassion”).

### 2.2. Eligibility Criteria

We included only studies where researchers analyzed quantitative data of effects of contemplative practices on trauma recovery. Given that our aim is to evaluate the role of contemplative practices in trauma recovery, focusing on and comparing specific characteristics, we excluded studies that used only qualitative data. The articles were restricted to those published in English and within the field of psychology, specifically excluding books, book chapters, and dissertations. Additionally, articles that were not peer-reviewed or that were published in languages other than English were excluded. After the initial search, two inclusion criteria were added: (1) a publication data after 1st January 2018 to limit the time span, given the presence of a previous review on earlier studies (i.e., [46]); and (2) the study design, including both non-randomized clinical trials and randomized controlled trials (RCTs).

### 2.3. Data Extraction

**Figure 1:**
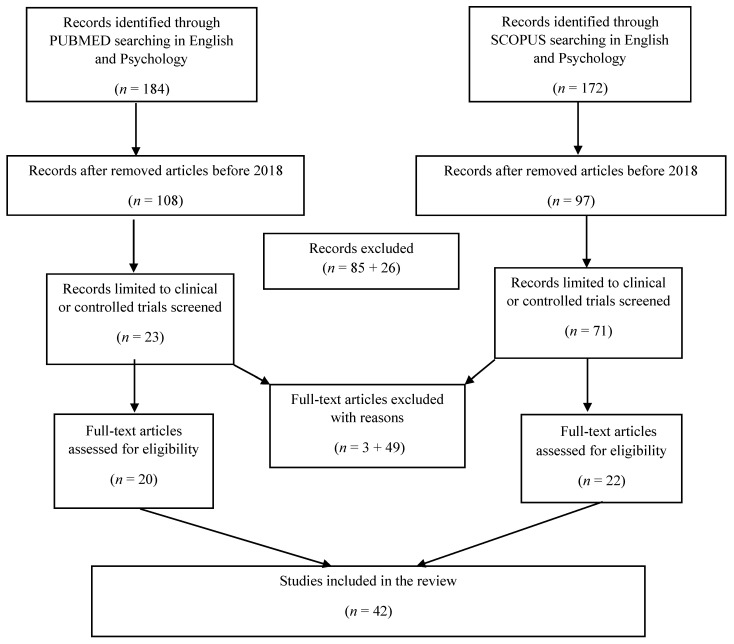
Flow diagram of the review process.

Data were initially extracted from the included studies by one reviewer (F.S.). Two additional reviewers (G.O. and A.L.) independently verified the accuracy of the extracted information. The extracted data included the type of contemplative practices, traumatic experiences/events or disorder, study sample, study design, measurements, and outcomes (Figure 1). Several secondary outcomes were tracked, but only those correlated with the effects of contemplative practices were reported. A summary of main results is illustrated in Table 1, which includes sample size, type of trauma, type of contemplative practices, duration, and main results. For further details of studies reviewed, especially regarding sample size, duration of intervention, and measures for primary and secondary outcomes, see the Supplementary Material (Table S1).

## 3. Results

### 3.1. Study Selection

The database search yielded 375 records. After the removal of duplicates, 209 titles and abstracts were screened. A total of 94 records were retained for full-text screening. After screening the full texts of the studies selected, 52 records were excluded based on the inclusion criteria. Specifically, the excluded studies relied on qualitative data, did not consider trauma-related measures as outcome variables, or treated mindfulness as a dispositional trait rather than as a psychological intervention. Overall, 42 studies were included in this narrative review (see Figure 1).

### 3.2. Study Characteristics: Design and Samples

Regarding the study design, the majority of selected articles were based on RCTs. These included active control (1, 5, 7, 10, 12, 13, 14, 16, 17, 19, 21, 22, 23, 24, 26, 27, 29, 30, 31, 33, 37) or a waitlist control group (2, 11, 15, 20, 36, 39, 42), single-group design study (3, 4, 6, 8, 9, 18, 25, 28, 34, 35, 38, 40), and collective case study (41). Half of the studies included multiple assessments with follow-up from one to twelve months (2, 4, 9, 10, 11, 15, 16, 18, 19, 20, 21, 23, 24, 25, 26, 28, 29, 31, 34, 36, 38).

The active control groups were based for instance on psychoeducation and low-intensity CBT skill training, relaxation techniques (12), or group training in integrated coping skills (ICS) (17). Other control groups related to informal activities were based on exchanging daily life experiences and playing board games (7) or multisport activity (21). Other examples of active control groups were PTSD intervention for problem solving and symptom management, the so-called Present-Centered Group Therapy (PCGT) (6), enhance usual care control (ETU) (24), and wellness control group, which included information on different aspects of health (5).

The intervention duration in the selected studies spanned from one 1,5 h session for the shortest intervention (32) to 36 sessions in 12 weeks for the longest interventions (2, 15). On average, the duration consisted of almost 11 weekly sessions. It is worth noting that positive outcomes were associated with interventions with average duration, while no significant difference from baseline or from the control group applies to short-time interventions. For instance, in a 6-day program combining scuba diving with mindfulness exercises (5), no significant changes in the total PTSD symptoms were reported but only in intrusion symptoms, and in the shortest study, the heart rate response increased contrary to the expected decrease (32). The association between duration of formal mindfulness practice (i.e., minutes for practice) and PTSD symptoms was explicitly found in a pilot RCT study at 6-month follow-up, suggesting that the longer the intervention was, the more avoidance, arousal, and reactivity symptoms were reduced (8).

Most samples were composed of a WEIRD population. A couple of studies targeted Black adults or partly included Black adults (16, 21). In nine studies, samples included veterans with PTSD symptoms (2, 6, 13, 15, 19, 28, 31, 32, 38). Other studies focused on samples composed entirely of women (7, 8, 17, 18, 20, 21, 29, 41), of which two studies (8, 18) specifically selected women with PTSD symptoms and substance abuse disorder. Interestingly, only three studies (11, 12, 36) selected asylum-seekers with forced displacement trauma and exposure to correlated collective stressors. Four studies targeted adolescents and young adults (35, 36, 37, 39), while the others recruited adults. Several interventions required adaptations since they targeted populations with specific sociocultural conditions (2, 10, 11, 12, 15, 16, 21, 24, 35, 36, 39), and one study also involved a program for Christian Church attenders (33).

### 3.3. Type of Trauma

The most common trauma investigated was PTSD from combat among male veterans (2, 6, 13, 15, 19, 28, 31, 32, 38). Other common traumas included PTSD related to comorbid substance use disorder (8, 17, 18, 24), violence such as intimate partner violence (21, 41), domestic violence (29), and physical, sexual, and emotional abuse (9, 27, 34) among women. Additional traumas included forced displacement and multiple traumatic experiences correlated with migration (11, 12, 36).

Three studies explicitly refer to a larger definition of trauma, called complex trauma (C-PTSD), also associated with multiple childhood trauma (20, 34, 38). Those traumas specifically regarded abuse, the threat of survival, experiencing helplessness, and fear or horror for witnessed or directly suffered violence early in life.

### 3.4. Type of Intervention

#### 3.4.1. Contemplative Practices as Single Interventions

In light of the call for novel mental health interventions and given the severity of large-scale mental problems, contemplative practices were investigated as cost-effective and feasible interventions for traumatic symptoms in comparison with more conventional therapies, such as Narrative Exposure Therapy (NET; [88,89]); individual psychotherapy, CBT [90], psychosocial interventions [91], Problem Management Plus (PM+), and e-health Self Help Plus (SH+; [92]).

The most evident result from the final dataset is that mindfulness meditation is the most common contemplative practice investigated in the last five years in association with trauma. Traditional programs Mindfulness-Based Stress Reduction (MBSR) were administrated alone (4, 13, 21), targeting veterans (13), women who experienced intimate partner violence (21), and police officers with occupational stress (4). A fourth study examined the effects of trauma-adapted intervention with explicit references to loving-kindness meditation and MBSR (9). Other studies combined mindfulness with cognitive–behavioral treatments (1, 6, 24, 27, 36), with trauma-adapted intervention from loving-kindness meditation (9) or with programs based on cultural adaptations (16, 36).

Seven studies involved constructive practices, such as compassion and loving-kindness meditation (6, 9, 12, 28, 30, 32, 34), delivered as structured programs. Only one study evaluated the effects of a single audio-taped session based on compassion meditation (LKM-S) on physiological recordings, where participants were asked to direct loving/friendly feelings toward themselves and others (32). Considering the relevance of the concordance between behavior and values in recovering psychological well-being, a study examined the role of acceptance and commitment therapy (ACT) in patients with distress due to the diagnosis of cancer (14).

Recent empirical evidence showed that ACT as mindfulness-based psychotherapy has beneficial effects on trauma-related symptoms [93].

One intervention, called Cognitively based Compassion Training (CBCT), targeted veterans with PTSD (6) and combined present-moment practices (i.e., focused attention and open monitoring) with analytical contemplative methods, which encourage cognitive reappraisal and alteration of usual mental patterns to expand compassion.

Three studies examined the effects of transcendental meditation (TM) on traumatic symptoms (29, 31, 39). For instance, they assessed the effectiveness of a standardized mantra-based form of meditation that enables a person to drift into a psychophysiological state of restful alertness (29).

The only movement-based contemplative practice investigated in the last five years in association with trauma is yoga (3, 7, 22, 35, 40, 41). A Kripalu yoga program combines physical postures, breathing, and moving meditation (7, 42), while a Hatha yoga program is an alignment-focused practice always linking breath with movement (3). Like with mindfulness, it was adapted for trauma conditions, generating programs such as the so-called trauma-informed yoga (35) and trauma-sensitive yoga (22, 41). In trauma-informed yoga, yoga forms were used as a tool for self-exploring various body movements, promoting physiological safety and calmness and helping to reorient the perceived danger, which could improve the ability to manage social relationships [23]. Trauma-sensitive yoga (TSY) was a structured body-oriented practice to discern curative elements of yoga that can enhance the healing of complex trauma by cultivating self-regulation, self-awareness, and nourishing a compassionate relationship with the body (40). Three principles of TSY to facilitate recovery for women with intimate partner violence include creating a safe environment to develop a sense of safety, consistency, non-judgment, and gentleness; only verbal assists are used if demonstrating respect for their physical boundaries; invitatory language.

No clinical trials examined the effects of tai chi and qi gong on trauma recovery in the last five years.

#### 3.4.2. Combined Therapeutic Interventions

Several programs in trauma conditions combined more standard treatments, such as cognitive therapy groups for trauma (16), cognitive behavioral therapy (36), trauma-adapted relapse prevention (3, 8), trauma and the body group (20), and internal family system therapy (34) with different types of meditation that included a variety of techniques. The combination of contemplative practices with trauma-related issues required training on strategies to manage flashbacks and the overload of intense emotions, such as the use of a deep breath as an anchor, placing hands on one’s belly (to have a physical reminder of breathing), and breathing until the response fades away. Moreover, this combination also emphasized the importance of a group setting, rather than individual one, as the group dynamic helps to reinforce the learning of meditation skills and enhances compassion by exposure to others’ experiences shared in the class. Group sharing focused on the difficulties and positive outcomes of the meditation learning process, rather than the content of traumatic experiences and symptoms, which could often evoke uncomfortable feelings. Over time, as trust developed within the group, participants felt more comfortable sharing their distressing experiences and coping strategies, creating a foundation for trauma-focused psychotherapy, although this was not the initial focus of the practice.

More integrated programs were an intensive treatment program (ITP), where yoga was combined with cognitive processing therapy (CPT), mindfulness, psychoeducation, art therapy, and integrative exercise (IE) (38). Integrative exercises included aerobic and resistance training exercises (2, 15).

Yoga and mindfulness-based cognitive therapy (Y-MBCT) were both included in the program named Trauma Intervention Mindfulness Based Extinction and Reconsolidation (TIMBER), aimed to allow for the cognitive reprocessing and neutral/detached reappraisal of the trauma memories. Furthermore, this program was the only one to be combined with a single sub-anesthetic dose of ketamine, chosen for its efficacy in the treatment of depression and PTSD but also for its ability to induce neurogenesis and to affect synaptic plasticity (27).

For childhood trauma complex PTSD symptoms, the trauma and the body group (TBG) method was developed as a group psychotherapy that combined mindfulness with sensorimotor psychotherapy. This included breath exercises, somatic check-in, discussions on home practice, brief mindfulness exercise, and psychoeducation on the impact of trauma on body weekly exercises (20). Furthermore, another program targeting complex PTSD symptoms for multiple childhood trauma was the Internal Family System therapy (IFS). This approach integrated systems theory and trauma theories with mindfulness, self-compassion, and self-acceptance practices (34).

Meditation was integrated into culturally adapted cognitive behavioral therapy (CA-CBT), which also involves psychoeducation, stretching exercises, and problem solving (e.g., management of social problems, motivational barriers and dissociation), targeting refugees with multiple trauma pre–post displacement (36). Similarly, mindfulness-based cognitive therapy (MBCT) was adapted for trauma exposed Black adults, also including psychoeducation and feedback and supportive group discussion of exercises beyond the skills training and in-class practice in mindfulness techniques (16). Regarding migration and displacement trauma, beyond CA-CBT, another specific program, the Mindfulness-Compassion Based Trauma Recovery for Refugees program (MBTR-R), was analyzed. The program was delivered in a group with a similar structure to MBSR and MBCT aiming to train in formal and informal mindfulness practices (e.g., body scan, sitting meditation, mindful movement, 3 min breathing space, formal and informal loving-kindness, and self-compassion practices). It also included psychoeducation on trauma-related mental health problems in refugees, trauma-sensitive themes (e.g., safe place practice), and sociocultural adaptations (e.g., separate groups for men and women, socioculturally specific metaphors) (11, 12).

Contemplative practices integrated with trauma-based interventions also targeted comorbidities and gender differences. Indeed, people with post-traumatic disorders often report symptoms of substance use disorder (SUD; [94]). Low levels of acting with awareness, a facet of mindfulness skill, for instance, may worsen trauma symptoms after emotional, physical, and sexual abuse (ETE), which, in turn, may lead to more craving for women in substance use treatment. A program especially designed for women with PTSD, called Trauma-integrated Mindfulness-Based Relapse Prevention (Ti-MBRP) was also a gender-responsive program and included gender themes, such as interpersonal relationships, parenting, and women empowerment, together with trauma themes, such as blame, overgeneralization of perception of threat to safe situations, and strategies to deal with hyperarousal (8, 17, 18). It also consisted in practices designed to bring awareness to cognitive and behavioral processes underlying substance use, reduce initial reactivity and promote non-judgmental acceptance, and explore substance use as a mechanism to cope with PTSD symptoms.

Another intervention targeting comorbid addiction was called intervention for dual problems and early action (IIDEA). It draws from a combination of cognitive–behavioral therapy and mindfulness, with more emphasis on cultural factors, motivation, and therapeutic alliance, considering the difficulties of building a trust relationship with these types of beneficiaries. It also includes psychoeducation, cognitive restructuring, and substance use recovery skills (24).

As part of preventive programs addressing occupational stress, a curriculum designed for police officers was inspired by the Mindfulness-Based Resilience Training Program. This curriculum included both formal and informal homework practices, body scan, movement, breath awareness, mindful eating, and compassion practice. Additionally, it placed a special emphasis on building resilience to stress and promoting neuroplasticity (4).

Among all the programs, two were fully delivered online. The mobile and telephone mindfulness program required recordings and homework on awareness of breathing, external stimuli (e.g., sound), emotions, and mindful acceptance (17). The other study was a game-based meditation intervention (MUSE) that utilized neurofeedback and included relaxation tutorials (e.g., deep-breathing techniques) followed by 3 min meditation sessions (37). After each meditation session, the participants were provided with feedback on their performance in the form of points and awards that reflect participants’ capacity to regulate their arousal.

The MUSE program, along with Learning to Breathe (L2B) were the only two programs delivered for adolescents with chronic stress (26, 37). It is particularly important to underline that online mHealth technology such as the MUSE program, which was delivered online (e.g., smartphone apps), may have side-effects, since meditation adverse effects are more likely to occur when practicing alone and without a qualified instructor.

Unlike MUSE, L2B was an in-presence program that combined mentoring and mindfulness. Its effectiveness was compared with a mentoring as-usual program, but it lasted just four sessions (26). Finally, only one study reported a personalization of mindfulness interventions on subject’s scores on Assessment Scale for Mindfulness Interventions (ASMI, clinician-rated) (27) while a second study included an individualized program of CBT-based treatment for depressive disorders and PTSD (30).

### 3.5. Effects of Contemplative Practices on Trauma Recovery

The primary finding regarding the effectiveness of contemplative practices was a significant reduction in PTSD symptom severity. For example, an average reduction of 31 points observed on the total CAPS score, a measure of PTSD symptom severity, was observed. This reduction met or exceeded the outcomes reported in other trials of veterans with PTSD for empirically supported psychotherapies, such as cognitive processing therapy or prolonged exposure therapy (15).

The impact on PTSD was frequently associated with a remarkable reduction in symptoms of depression and anxiety. With only a few exceptions (10, 36), in most cases (1, 4, 6, 7, 11, 12, 19,28, 29, 31, 38, 39), depression, anxiety, stress, and PTSD symptoms were highly correlated and decreased together after the intervention. In a sample of veterans (10), the mind–body skills group reduced PTSD symptoms but had no significant effects on depression and anxiety. Conversely, culturally adapted cognitive–behavioral therapy (CA-CBT) reduced depression and somatic symptoms in refugees but did not result in significant changes in the PTSD checklist (36).

Data from physiological parameters support the above results: a first study (13) reported an increase in frontal theta heartbeat-evoked brain response (HEBR); other studies reported lower basal activity of the sympathetic nervous system (37) and cortisol levels (40). A counterintuitive result was revealed in a fourth study that showed lower Skin Conductance Levels (SCLs) (meaning a reduction in sympathetic arousal), but contrary to what expected, a higher heart rate response was observed (indicating higher physiological arousal), and a not-significant change in heart rate variability followed the intervention (32).

It is interesting to note that programs may have different impacts on several dimensions of PTSD symptoms. For instance, a study on a program that combined mindfulness with scuba diving had a non-significant overall effect on PTSD symptoms, except for the subscale of intrusion, which was significantly reduced at post-test and at first follow-up (5). The subscales of intrusion together with avoidance were also reduced after Kripalu yoga, while a not-significant effect was observed on the hyperarousal subscale (7). Avoidance was the dimension most notably affected by the trauma-adapted intervention from LKM and MBSR, with an effect that persisted at follow-up (9). A mind–body skills group affected avoidance and hyperarousal, together with sleep disturbance and anger (10). Other studies showed a decrease in all the subscales: intrusions, avoidance, negative changes in cognitions and mood, and changes in arousal and reactivity (e.g., 1).

Regarding follow-up studies, some research found no significant effects at follow-up; for instance, neither formal nor informal practice predicted a reduction in intrusion symptoms and craving at 6-month follow-up in women with SUD and PTSD after MBRP (8). No significant effects were found either for overall PTSD score or mindfulness at 3-month follow-up after a brief program of mindfulness and scuba diving (5). Similarly, Hatha yoga did not produce significant effects on intrusion and avoidance symptoms at follow-up in people with comorbid chronic pain (3). On the contrary, other studies found effects of MBSR on PTSD symptoms at 5-month follow-up (4) up to a one-year follow-up (36). This latest intervention, named culturally adapted cognitive–behavioral therapy (CA-CBT), increased emotion regulation skills, somatic symptoms, quality of life, and general health, with main effects maintained at one-year follow-up in migrants with multiple displacement trauma (36).

Regarding the effects of the two programs fully delivered online, it is worth noting that the mobile program showed higher level of drop-out in comparison with the telephone program, and no significant effects on mindfulness and coping orientation were observed (25). The MUSE program based on game meditation in adolescents showed a reduction in sympathetic nervous system basal activity, but there was only an increase in hypothalamic–pituitary–adrenal axis reactivity, without a significant change in autonomic nervous system reactivity to acute stress (37).

Other significant changes associated with contemplative practices regard positive dimensions of well-being, social feelings, and behaviors. Fewer studies reported outcomes of the effectiveness of the interventions on psychological well-being in eudaimonic or hedonic aspects rather than measures of symptoms and psychopathology. MBSR for occupational stress in police officers reduced operational stress, the exhaustion subscale of burn-out, and negative affect, but it did not significantly influence positive affect (4). It also increased eudaimonic well-being, as measured with the Psychological Well-being Scale (4). Other interventions showed positive effects on health responsibility, stress management, not feeling dominated by symptoms (19), life satisfaction (40), and quality of life (2, 9, 15, 28, 36). Studies especially focused on compassioned intervention reported increases in self-compassion (9, 11, 32). A study that delivered Internal Family System therapy did not show significant increase in self-compassion after the intervention in comparison to controls while revealing effects on PTSD symptoms and psychological distress (34).

Regarding results on social domains, studies reported an increase in social connectedness (6) and social role functioning (3), and on the other hand, they reported a decrease in fear of compassion, feeling of self-inadequacy, and levels of external shame (28). Compassioned mind training targeting not only ex-service personnel of defense forces with PTSD but also their female partners evaluated positive relational outcomes, such as social safeness and relationship satisfaction (28). Only one study investigated and revealed positive effects of the treatment on improvements in values-consistent behavior (BEVS) after an intervention based on acceptance commitment therapy for cancer survivors (14).

As for emotion regulation in combination with PTSD symptoms, two studies (21, 26) reported a decrease in the Difficulties in Emotional Regulation Scale, one study (36) reported an increase in emotion regulation (ERS), and one study (6) reported no significant changes, as measured by the Differential Emotional Scale. Theoretically correlated to emotion regulation, there is the soothing receptivity, which is the experience of being soothed physically, capacity to be soothed, experiencing soothing by disclosing to others, and self-soothing [95,96,97]. Soothing receptivity, often missed in traumatic victims, was strongly increased after the mind–body group therapy (TBG) applied on childhood complex trauma (20). Indeed, this type of therapy allowed participants to engage in both self-soothing and relational soothing, giving them the opportunity to experience their bodies and others not as a source of hurt any more but as a place of healing.

Research on contemplative practices in trauma recovery also assessed the effect on a relevant correlation of emotion regulation, which is interoceptive ability as measured by the Scale of Body Connection (SBC) (20) or by the MAIA (34). It is interesting to note that the above-mentioned program led to higher awareness of somatic experience, i.e., the body-awareness subscale of SBC, but did not significantly affect the bodily dissociation subscale of SBC and the Dissociative Experiences Scale (DES) (20). Regarding MAIA, a large positive effect on the dimension of trusting, a medium effect on attention regulation, self-regulation, and body listening at post-test, and an increase in not-distracting at 1-month follow-up after the intervention (IFS) were revealed in an uncontrolled clinical trial study (34); a significant increase in emotional awareness, self-regulation, and body listening in an RCT with multiple assessments after integrative exercises (IEs) was also observed (15). Self-regulation, that is, the ability to regulate psychological distress by attention to body sensations, was the largest effect size revealed in parallel with the reduction in hyperarousal on the PTSD symptoms and the increase in the mindfulness facet of non-reactivity, which denotes the tendency to allow thoughts and feelings to come and go without getting caught up in or carried away by them (15).

Interestingly, a collective case study design provided data from interviews (pre- and post-intervention), analyzed through content thematic analysis (41). Trauma recovery benefits were conceptualized as body sensitivity, emotional benefits such as confidence and peace, and improvement in social relationships. After participating in trauma-sensitive yoga, individuals reported several key themes regarding its benefits. The first theme highlighted improvements in physiological functioning, including better sleep, enhanced body attunement, and a greater sense of relaxation. Other reported physical benefits were the release or reduction in tension and pain, increased physical strength, improved balance and alignment, and increased attention to muscle soreness. Participants also described heightened sensitivity to physical warmth, visceral sensations, and overall boosts in energy. A second theme centered on emotional benefits: participants experienced increased feelings of peace, pride, confidence, and hopefulness, along with greater self-permission and self-acceptance. Simultaneously, they noted a reduction in negative feelings, such as anxiety and irritability. Responses to previously triggering events became calmer, suggesting a reduced perception of threat in safe conditions. A third theme focused on enhanced and more stable interpersonal relationships. Participants reported increased trust in others, as they wished in their pre-interviews, and they felt better able to navigate challenging interactions with family members because they were calmer and more connected. Additionally, they highlighted benefits stemming from a commitment to self-care and a sense of accomplishment in completing tasks, such as attending and finishing the TSY classes.

A study assessing the feasibility and acceptability of a culturally adapted MBCT (16) identified several key benefits through narrative analysis. Participants frequently reported improvements in awareness and attention, enhanced emotion regulation, and better coping skills to manage stress or depression. Additionally, benefits included an increased sense of control, energy and positive affect, greater relaxation and calm, experiencing less physical pain, and fewer interpersonal conflicts. The group format also provided valuable peer support, which participants found beneficial. In addition to the reported benefits, the study also examined the barriers to participation in the treatment. The most common challenges were related to physical and transportation issues, which impacted attendance. Other barriers included stigma, lack of motivation, no availability of services, emotional concerns, misfit of therapy to needs, and time constraints. According to qualitative reports, more than half of the respondents expressed that the cost of treatment was too high to be sustainable over time.

In a mixed-methods study, participants with double diagnoses (substance abuse and chronic stress) found the following to be useful: being listened to without judgement, learning relaxation and emotional regulation techniques, gaining a sense of self-control, and managing the double-diagnosis symptoms (24). The study complemented previous research [48], which revealed quantitative effects on physiological tests, such as urine tests, therapeutic alliance, mindfulness, illness management and recovery, depression, anxiety, and PTSD symptoms.

Regarding co-occurring substance use disorder, it is worth noting that a study reported the effectiveness of interventions incorporating contemplative practices for certain PTSD symptoms, specifically avoidance, arousal, and reactivity. However, these interventions were not as effective for addressing symptoms related to intrusion and re-experiencing (8). Another study by the same authors showed a reduction in cravings and PTSD symptoms after the two programs (MBRP and Ti-MBRP) compared over the 12-month follow-up period, with effect sizes similar to other PTSD-SUD interventions but with a high level of attrition and a high level of retention that was lower than expected (18). The level of completion of the program in double-diagnosis patients depended on the PTSD symptom score; participants with higher symptom severity were more likely to drop out in both groups (17). Conversely, a higher level of completion occurred in women in the experimental group with lower PTSD symptoms and higher mindfulness scores, as measured with the FFMQ (3). A clinical trial with veterans showed a large effect size on hyperarousal, reexperiencing, negative alterations in cognitions, and depression but no significant effects on positive and negative emotions (DES) nor in alcohol consumption (6).

Few studies pointed out a process analysis to understand how the interventions affected the outcome variables, PTSD symptoms. Specifically, two studies revealed the mediating effects of self-criticism and shame (12, 14) in the relationship between treatments (respectively, the Mindfulness-Compassion Based Trauma Recovery for Refugees program (MBTR-R) and acceptance commitment therapy) and PTSD symptoms, while a study focused on the change in PTSD symptoms not just by the decrease in self-criticism but also by the increase in self-compassion (11). A study focusing on mediation analysis showed that acceptance and commitment therapy (ACT) led to an increase in self-compassion and coping strategies of emotional processing and expression. These improvements, in turn, contributed to a reduction in cancer-related trauma symptoms. Additionally, self-compassion, emotional approach coping, and values-aligned behavior marginally mediated improvements in fear of recurrence and general anxiety symptoms (14).

A study on electrophysiological indicators revealed that frontal theta heartbeat-evoked brain responses (HEBR) was a mediator in the relationship between MBSR and PTSD symptoms. Hence, the intervention reduced PTSD symptoms through an increase in frontal theta HEBR (13). Finally, a fifth study found mindfulness, in particular, the Positive States of Mind Scales and the subscale Non-Reactivity of FFMQ, to partially mediate the treatment effect on hyperarousal (CAPS subscale) and psychological quality of life (15).

A study examining moderator factors found that the level of adult interpersonal experiences influenced the relationship between treatment and PTSD symptoms (22). Indeed, the trauma-sensitive yoga intervention was more effective for people with a history of less adult-onset interpersonal trauma experiences. Depression, dissociative symptoms, and affect dysregulation were not moderated by experiences of adult interpersonal trauma in the TCTSY intervention. Furthermore, all pre-intervention measures, except the pre-test depression score (dissociative symptoms, affect dysregulation, emotional control problems, PTSD symptoms), moderated intervention condition effects for participants exposed to fewer (i.e., one) forms of adult interpersonal trauma. In summary, the overall results indicated that the efficacy of the intervention conditions was less predictable among those with more interpersonal trauma experiences.

Finally, only two studies pointed out the adverse effects of the programs. A study on transcendental meditation reported twelve mild adverse events, as reported by six participants (i.e., nausea, headache, irritability, weight gain). Two participants self-reported a severe adverse event that they believed was related to the intervention (i.e., cold-sores, body feeling heavy) (29). A second study on trauma-informed yoga reported psychological effects, as reported by just a few respondents, such as feeling upset, anxious, or stressed after class. It is interesting to note that the group involved in substance use treatment, which retrospectively reported feeling upset, anxious, or stressed before the yoga class, was also the group that showed the lowest levels of change in self-regulation skills (35).

### 3.6. Measures

#### 3.6.1. Primary Outcome

Except for the Clinician-Administered PTSD Scale for DSM-5 (CAPS-5) (2, 6, 9, 13, 16, 17, 27, 21, 24, 41, 42), self-report measures are the prevalent type of assessments. Self-reported PTSD symptoms and traumatic events were measured using a variety of questionnaires: the PTSD Symptom Scale Self Report PSS (17); the Davidson Trauma Scale (DTS) (9, 34); the Structured Interview for Disorders of Extreme Stress-Self-Report (SIDES-SR) (34); the Harvard Trauma Questionnaire (HTQ) (12); the Traumatic Events Inventory (TEI) (16); the Impact of Events Scale-Revised (IES-R) (7, 42); the PTSD Checklist for DSM-5 (PCL-5) (5, 6, 8, 13, 20, 21, 27, 28, 29, 31, 32, 36, 38); the Childhood Trauma Questionnaire (CTQ-SF) and the Life Stressor Checklist Revised (LSCR-R) at baseline for recruitment (20); the Intimate Partner Violence Exposure (IPVE) and Life Events Checklist (LEC-5) at baseline for recruitment (21); the Child PTSD Symptom Scale on Distressing Events (26); the PTSD Checklist–Civilian (PCL-C) (4, 30, 39, 42); and the Trauma History Questionnaire (39).

CAPS-5 is the most-used measure, which is a 30-item structured interview that corresponds to the DSM-5 criteria for PTSD, considered the gold standard in PTSD assessment, and it is often used in combination with PCL, the PTSD checklist. For the assessment of DESNOS, the measure used was the Structured Interview for Disorders of Extreme Stress—Self-Report (SIDES-SR), while for developmental trauma disorder and multiple childhood trauma, measures included the Child PTSD Symptom Scale on Distressing Events, a self-report scale that assesses PTSD symptoms in 8–18-year-old children who have experienced a traumatic event; and the Childhood Trauma Questionnaire-Short Form (CTQ-SF), a self-report questionnaire that assesses several types of childhood abuse and maltreatment in adults.

#### 3.6.2. Secondary Outcomes

Other specific symptoms measured through self-report assessments include depression through the Patient Health Questionnaire PHQ-9 (6, 11, 12, 13, 16, 25, 30, 32, 36) PHQ-8 (38), PHQ-15 (13), the Beck Depression Inventory (BDI) (9, 20, 31, 34, 39), the clinician-administered Hamilton Depression Rating Scale (Ham-D) (20), the Depressive Experiences Questionnaire (28); the Depression, Anxiety and Stress Scale (DASS-21) (7, 28), the Hospital Anxiety and Depression Scale (HADS) (14); anxiety through the Beck Anxiety Inventory (BAI) (20, 27, 31), and the Generalized Anxiety Disorder Scale (GAD-7) (24).

Dispositional mindfulness was measured using the Five-Factor Mindfulness Questionnaire (FFMQ) (9, 15, 16, 17, 26), Freiburg Mindfulness Inventory (FMI) (5), Philadelphia Mindfulness Scale (PHLMS) (6, 20), Mindfulness Awareness Attention Scale (MAAS) (24), and the Cognitive and Affective Mindfulness Scale—Revised (CAMS-R) (25), all self-report assessments.

In some studies, emotion regulation capability associated with trauma and interventions was measured using self-report questionnaires such as the Difficulties in Emotional Regulation Scale (DERS), which includes non-acceptance of emotional responses, difficulties engaging in goal-directed behavior, impulse control difficulties, lack of emotional awareness, and limited access to emotion regulation strategies (17, 21, 26, 36), the Emotion Regulation Questionnaire (ERQ) (32), and the Differential Emotion Scale (DES’) (6). Also, two studies measured interoception using the Multidimensional Assessment of Interoceptive Awareness (MAIA) scale (15, 34).

In addition, it is worth mentioning that a few studies included physiological parameters to measure brain waves in resting meditation and heartbeat-evoked brain responses (HEBR) through EEG and ECG (13); heart rate variability (HRV) (32) and basal ANS activity (37) through ECG; skin conductance levels (SCL) (32); HPA axis activity through hair (37); and salivary cortisol levels (40).

Finally, combined with quantitative results, some studied added qualitative and explorative methods, especially aimed at evaluating the feasibility and acceptability of the intervention or applying the subjective meaning of trauma recovery. These studies reported what participants felt were benefits from the treatment and developed multiple interpretations that optimized an understanding of trauma recovery (16, 24, 41).

## 4. Discussion

Do contemplative practices promote trauma recovery? Most of the reviewed studies showed significant improvements in PTSD symptoms. Specifically, contemplative practices have proven to be effective in reducing most dimensions of trauma, including avoidance, reactivity, intrusion, hyperarousal, and negative cognitions and mood. Nevertheless, although this review revealed that contemplative practices improve PTSD symptoms overall, it remains unclear whether these interventions may reduce all specific facets of post-traumatic disorder. If some studies revealed changes in all the dimensions [21], other studies reported discrepancies in the effects of contemplative interventions. For instance, Goldstein et al. [47] investigated the effects of a group-based integrative exercise (IE) program on PTSD symptoms in a group of military veterans, finding that participants in the IE group did not report improvements in symptoms of re-experiencing and avoidance when compared with the control group. Similarly, Chopin et al. [48] tested the efficacy of a yoga program among military veterans, finding that re-experiencing and avoidance PTSD symptom subscales did not improve after treatment. Grupe et al. [49] examined the effects of mindfulness meditation among police officers, finding large reductions in hyperarousal immediately after training and at 5-month follow-up, reductions in re-experiencing immediately after treatment but not at follow-up, and no changes in avoidance symptoms at either time point. Gibert et al. [50] examined the effects of a diving program called the Bathysmed^®^ protocol on PTSD symptoms, reporting that, except for intrusion dimension, none of the other facets changed after treatment; moreover, the effects on the intrusion subscale did not persist at three-month follow-up. Lang et al. [51] found that a Cognitively Based Compassion Training did not have significant effects on the avoidance subscale in a group of military veterans. Differently, Yi et al. [52] reported that the intrusion and avoidance subscale, but not hyperarousal, was significantly reduced in women with PTSD immediately after a Kripalu-based yoga intervention and at 3-months follow-up. Somohano et al. [53] examined the effects of a mindfulness intervention among women with PTSD, reporting that a higher duration of mindfulness practice predicted lower avoidance, arousal, reactivity, negative cognitions, and mood but did not predict improvements in the intrusion subscale at six-month follow-up. Muller-Engelmann et al. [54] found that the avoidance dimension was especially reduced by trauma-adapted intervention from LKM and MBSR, keeping its effect at follow-up. Similarly, Staples et al. [55] found that a mind–body skills group especially reduced avoidance and hyperarousal. In sum, integrative exercise (IE), a general yoga program, and Cognitively Based Compassion Training did not affect avoidance, differently from the Kripalu-based yoga intervention, trauma-adapted intervention from LKM and MBSR, and mind–body skills group. Mindfulness meditation had different outcomes on the avoidance dimension. This discrepancy may, in part, be attributed to the type of intervention administered. Many treatments that did not significantly impact the dimensions of avoidance and intrusion consisted solely of contemplative practices without incorporating trauma-based interventions. Indeed, avoidant behaviors, which are often triggered by adverse events reminiscent of traumatic experiences, and the re-experiencing of traumatic memories (i.e., intrusion) are particularly challenging symptoms to address. These symptoms may require a psychotherapeutic approach specifically designed for trauma and an extended duration of treatment to achieve meaningful improvements. This result is in line with the existing literature that indicates that interventions explicitly focused on traumatic experiences—such as those incorporating trauma narratives—tend to be more effective than approaches that do not directly address trauma [98]. This can be one fundamental reason why contemplative practices do not appear to yield improvements across all dimensions of PTSD, suggesting that trauma-focused psychotherapies may be necessary to achieve more comprehensive outcomes.

The hypothesis of a dose–response effect could also be supported by some controversial results from physiological parameters (13, 32, 37, 40). Authors suggested that a longer intervention is needed to produce physiological changes (such as a change in heart rate variability), considering that shifting from threat to a soothing emotion system is not immediate [99]. At the same time, a longer treatment might be needed in order to increase a sense of security and reduce the threat related to aversive experiences and, consequently, avoidant behaviors that have a self-protective function.

Furthermore, physiological results showed a reduction in sympathetic arousal and lower basal activity of the SNS (sympathetic nervous system). The dysregulation of SNS together with parasympathetic disinhibition might be a marker of behavioral activation or inhibition [100], according to the neurovisceral integration model [101], and it is theorized to precede cognitive changes [102]. Moreover, the studies reviewed have shown that reductions in sympathetic activity during rest were associated with decreases in self-reported post-traumatic symptoms. This finding is in line with research indicating that lower levels of sympathetic activity are associated with decreased feelings of fear [103] and threat [104]. Future research could deepen the complex causality of the relation between physiological and psychological parameters in PTSD symptoms.

The reviewed studies highlighted the effectiveness of contemplative practices when combined with health or psychotherapeutic programs in both the prevention and treatment of post-traumatic stress disorder (PTSD). When integrated into clinical psychotherapies, contemplative practices emerged as important interventions for individuals with PTSD. For example, Structured Psychotherapy for Adolescents Responding to Chronic Stress (SPARCS; [105]) is a group trauma-focused program for urban adolescents that aims to enhance stress identification, awareness of bodily and emotional responses, mindful non-judgmental approaches, communication and problem-solving skills, self-soothing, and distress tolerance. The RAP Club, a trauma-informed program for eighth-graders, is based on SPARCS and similarly promotes self-regulation through psychoeducation, cognitive–behavioral therapy (CBT), and mindfulness strategies [105,106]. For adults with PTSD, including veterans, trauma-informed adaptations of contemplative practices have been developed. These include psychoeducation on trauma and stress physiology, fostering autonomy and a sense of control during exercises, and offering flexible practice options (e.g., allowing participants to keep their eyes open to prevent triggering fear or distress). Grounding exercises are incorporated at the start of sessions, and the duration of sitting meditations is often reduced to accommodate participants’ needs. Contemplative practices combined with trauma-informed and trauma-based psychotherapies have also been applied in educational and professional settings [107], targeting social workers [108], nurses, and other practitioners. These interventions support the management of vicarious trauma, compassion fatigue, and work-related stress while fostering emotional resilience and a sense of hope [109].

According to our results, mindfulness is the contemplative practice most used to promote trauma recovery. Mindfulness meditation is defined as paying attention to the present moment, being aware and non-judgmentally accepting [110,111]. Thus, several authors stated that mindfulness meditation is effective in reducing PTSD due to a top-down approach (e.g., [112]). Indeed, as mindfulness is focused on the ability to act with awareness and non-judgmentally, as well as on the capacity of reorientation of habitual patterns of thinking, it may be therapeutic for trauma-related rumination and reduce emotional distress and hyperarousal, even without a direct discussion of trauma-related content (e.g., [46]). Participants are encouraged to attend to arising and passing stimuli, including aversive trauma-related memories, increasing tolerance for experiencing negative affective states, and reducing numbing and hyperarousal when faced with trauma symptoms.

Interestingly, our findings revealed that contemplative practices also improved various secondary outcomes, including anxiety, depression, operational stress, negative affect, interoception, and emotion regulation. In most cases, reductions in PTSD were intuitively associated with the decrease in anxiety and depression. This is not surprising if we consider the literature on the effects of MBIs on internalizing symptoms [12,13,14].

Regarding interoception, interventions predicted positive effects almost on all the dimensions of the MAIA, revealing self-regulation to be the largest effect size in parallel with hyperarousal and non-reactivity (15). Interoception was also correlated to emotion regulation and soothing receptivity.

In addition to reducing distress-related psychological outcomes, findings showed the ability of these interventions to enhance eudaimonic well-being, social domains such as increase in social connectedness (6), social role functioning (3), and overall quality of life. Qualitative results found changes in participants’ search for life meaning, a narrative revaluation of life experiences, and a reordering of spiritual and universalistic values. Participants also reported shifts in time perspective, an increased attitude of self-care, and the adoption of positive coping strategies (24). These results recall previous qualitative studies that testify the so-called post-traumatic growth, which is often reinforced by formal and informal contemplative practices [112,113], in the aftermath of traumatic events. In addition, results seem to give support to Mindfulness-to-Meaning Theory (MMT; [114]), as mindfulness appears to facilitate the cognitive reappraisal of traumatic events and rebuild a new meaning and purpose in life. Previous results showed effects of MBI programs on eudaimonic well-being, especially on purpose in life in adolescents [14].

Another perspective on the effectiveness of these interventions concerns the specific diagnoses characterizing the study samples. For example, individuals with co-occurring SUD (substance use disorder) required more intensive mindfulness skills training either prior to or during treatment to enhance their willingness to experience distressing thoughts, feelings, or substance cravings. Previous studies supporting the efficacy of MBIs for managing craving highlighted several mediators of this effect, including reductions in negative affect as well as improvements in emotion regulation, emotional well-being, and group cohesiveness [115].

Regarding the process analysis, it is interesting to reveal that just five of the reviewed studies (11, 12, 13, 14, 15) carried out mediation analyses in order to examine the psychological or physiological mechanisms, which may explain the improvements in PTSD symptoms following contemplative practices. For example, Aizik-Reebs et al. [56] found that reduced levels of self-criticism as well as improved self-compassion following a Mindfulness-Based Trauma Recovery for Refugees (MBTR-R) program mediated the therapeutic effects of the intervention on PTSD symptoms. This result recalls similar previous results in a different population characterized by double diagnosis of SUD and PTSD, where contemplative practices predicted a reduction in shame and no-judgmental attitude, and these, in turn, affected substance use severity and PTSD symptoms [116,117]. In another study with MBTR-R, Oren-Schwartz et al. [57] found the mediating role of shame in the relationship between the therapeutic effects of MBTR-R and PTSD symptoms. Kang et al. [58] found that MBSR reduced PTSD symptom severity by improving the frontal theta heartbeat-evoked brain response (HEBR) in a group of military veterans with PTSD. Fishbein et al. [59] carried out a multiple mediation model wherein self-compassion and emotional coping mediated the relationship between acceptance and commitment therapy effects and cancer-related trauma symptoms among cancer survivors. Finally, Mehling et al. [60] found the mindfulness facet of non-reactivity and positive state of mind to partially mediate the treatment effect on hyperarousal and psychological quality of life.

These processes, which involved both psychological and physiological mechanisms, seem to be of relevance to reduce or improve the effects of contemplative practices on trauma recovery. In particular, contemplative practices physiologically increase heartbeat-evoked brain responses that are interoceptive signals, thereby improving attentional control and resting brain states. Interoceptive neural functions seem to be a primary cerebral mechanism that improves symptoms of PTSD, given that contemplative practices train to cultivate attention to the present moment and bodily sensations, reinforcing interoception. The somatic component of contemplative practices is pivotal in facilitating the trauma recovery process by allowing individuals to notice visceral feelings and forge somatic connections [118]. In fact, contemplative practices based on body movements and active breathing, which can be considered bottom-up interventions, could enhance interoceptive ability by triggering involuntary attention on the unblocked feelings that were repressed [30].

Another mechanism through which contemplative practices contribute to trauma recovery is their influence on fostering positive states of mind, particularly self-compassion vs. self-criticism and self-blame. After experiencing traumatic stressors, individuals often develop negative, self-critical appraisals and beliefs (e.g., “I am worthless/weak/wrong because I am not able to deal with it”). These self-critical patterns can perpetuate chronic stress over time [119]. In contrast, self-compassion—a multidimensional construct—offers an alternative approach. It encompasses self-kindness, recognition, and understanding the universality of suffering, tolerating it without judgement and committing to actions that reduce further suffering [120,121]. Contemplative practices actively cultivate these qualities, helping individuals replace self-criticism with self-compassion, which may alleviate the psychological burden of trauma and support recovery. It is reasonable to hypothesize that enhanced attention to the present moment, combined with the compassionate thoughts and feeling elicited by explicit self-compassion practices, transforms the way individuals relate to mental states such as rumination and self-blame. By bringing these states into awareness as objects of observation, contemplative practices reduce identification with negative thoughts and actively foster attitudes of self-kindness and common humanity.

A core goal of those programs is to strengthen specific mental states that help the engagement of the individual’s compassionate self, bolstering a safe internal environment that improves processing of traumatic memories and assist healing, involving curiosity, calm, clarity, connectedness, courage, and creativity [122]. Future studies could investigate the protective effect of self-compassion for the intrusive and pervasive thinking associated with traumatic experiences and amplify the psychological changes following contemplative practices.

## 5. Limitations

This review also points out some limitations. First, outcome data were collected using self-report measures, which can be susceptible to issues such as practice effects and response bias. Moreover, other factors, such as cohesion, treatment engagement, therapist characteristics, are not always available in the studies here selected and should be included as covariates in future research. Second, most studies are based on a small sample size. In clinical contexts, it is usual to have fewer participants in the trials, since the sample size should not be a limitation if we investigate our research questions with a more adequate methodology that fits with the sample and the aims. The lack of significant effects in some cases, indeed, could cover relevant effects that do not emerge because of the small sample size, while in some studies we assist to a variety of variables, too many investigated in a single study, that could overestimate significant effects on the other side. Hence, we argue that there is a problem of methodology and that clinical questions could be addressed more properly using qualitative methods for small scales, leaving more complex statistical models for larger samples (type II error). A third limitation is related to the uncertainty on the relationship between treatments and effects. This applies particularly to mixed interventions (e.g., mindfulness with trauma therapy), in which it is impossible to know exactly what produces the effects. Future studies could combine interviews and focus groups with quantitative studies in order to investigate what exactly produced a change in the participants according to their qualitative reports. Fourth, in most cases, the sample was composed of White participants living in an urban context. Although we are aware that white or WEIRD (Western, Educated, Industrialized, Rich, and Democratic) people are more likely recruited in these kinds of clinical trials because they have better access to contemplative practices, the lack of ethnic, cultural, and geographical heterogeneity in the sample limits its cross-cultural validity. It is unclear whether the findings would be translatable to others, non-WEIRD, living in non-metropolitan regions. Furthermore, the data show that the feasibility rates were lower in the treatment groups, emphasizing that barriers to engaging in a treatment exist and need to be addressed to improve outcomes. High drop-out in several studies make it difficult to interpret the results on the effectiveness and feasibility of the proposed interventions. In addition, most studies did not analyze the effects of interventions, comparing completers and non-completers. The retention issue is especially present for the non-WEIRD population due to psychosocial problems, material and financial reasons, and cultural barriers among others. Understanding barriers to retaining those in the experimental group is a key factor for future implementation. For instance, it is crucial to consider cultural differences in experiencing self-criticism as shame or guilt and in their impact on psychopathology and traumatic symptoms. In the WEIRD population, guilt could have a prominent role, having psychopathogenic or traumatogenic effects independent of shame [123]. Contrarily, among asylum-seekers and the general non-WEIRD population, shame, rather than guilt, seems to have a bigger impact on psychopathology and traumatic symptoms (11, 12). This may be due to the fact that the non-WEIRD population generally belongs to a more community-oriented culture, where shame is more associated with the fear that one’s problems will result in the loss of union with, or expulsion from, the group [124]. Some studies focused on alternative samples like groups of refugee population (11, 12), yet they pose a limit to the generalizability of findings considering their stressful, uncertain, and insecure urban post-displacement setting [125]. Nevertheless, this type of PTSD disorder victim represents a common and fast-growing population, given the global spread of wars, famine, climate disasters, and pandemics. It is, indeed, a limitation to find little attention to collective traumas. In this regard, future studies should overcome this gap in the literature, investigating more community treatments for collective traumas, given the global emergencies that we are dealing with such as the climate crisis with a huger number of victims of acute and chronic environmental stressors.

## 6. Conclusions

The present review showed the effectiveness of programs integrating contemplative practices in the treatment of PTSD victims. It also underlined controversial results and limitations that could be overcome in future investigations. In dealing with the fast-growing large scale mental distress, especially due to the emergent global collective traumatic events and conditions, contemplative practices could be cost-effective and feasible interventions for traumatic symptoms. Nevertheless, their overall effectiveness depends on the duration and the combination with trauma-focused treatments. Given the large variety in health programs for trauma, the authors do not call for novel interventions; rather, they suggest a combination of programs that integrate somatic components with cognitive and emotional ones. On one side, people with post-traumatic stress need support to learn to stay with the discomfort of emotions, thoughts, bodily sensations, the faint-freeze response to acute stress and the defensive mechanisms activated for the neuroperception of threat. On the other side, by allowing a neuroperception of safety and cultivating self-compassion attitude, interventions and practices can focus on emotions, bodily sensations, and thoughts as an opportunity to reframe past memories. If needed, they also can facilitate the completion of emotional action sequences that were interrupted or restrained in the past, when the traumatic events or relationships occurred. Contemplative practices could contribute to promoting this sense of safety, allowing psychotherapeutic interventions focused on trauma to work on reframing dysfunctional beliefs, emotion expression, and metacognitive processes in order to heal the sense of self, often wounded in post-traumatic victims.

## Figures and Tables

**Table 1:** Studies’ characteristics and main results.

N	Authors	Type of Trauma	Type of Contemplative Practices	Duration	Main Results
1	Jasbi et al., 2018 [21]	PTSD	MBCT + Citalopram	8 weekly sessions (60–70 min each)	↓ PCL-5 (re-experiencing events, avoidance, negative mood and cognition, hyperarousal); ↓ DASS (depression, anxiety, stress).
2	Goldstein et al., 2018 [47]	PTSD	IE	36 sessions in 12 weeks (1 h each)	↓ CAPS-5 total 31 point reduction at post-test; ↓ Subscale of hyperarousal; ↑ LSI (more physical activity); ↑ WHOQOL-BREF greater improvement in the psychological domain but a smaller improvement in the physical domain. Greater number of sessions attended was associated with an improvement in physical quality of life and psychological quality of life. High levels of satisfaction.
3	Chopin et al., 2020 [48]	PTSD with comorbid chronic pain	Hatha yoga	10 cohorts (2 to 8 weeks): 90 min each	↓ PTSD symptoms, kinesiophobia, depression, and anxiety. Follow-up results: ↔ Intrusion and avoidance symptoms; ↑ Social role functioning PROMIS.
4	Grupe et al., 2021 [49]	Occupational stress	MBSR	8 weekly sessions	↓ PSQ operational stress and moderated by gender and years of police experience: younger men showed greater decline in stress also at follow-up; ↓ PCL at post-test and at 5-month follow-up; ↓ Exhaustion subscale of OLBI; ↓ PROMIS anxiety symptoms and depression symptoms; ↓ PANAS negative affect; ↔ PROMIS subscales of pain interference; pain intensity, or physical functioning; ↔ Disengagement subscale of OLBI; ↔ PANAS positive affect; ↔ Physical parameters; ↑ Sleep quality PSQI; ↑ PWB.
5	Gibert et al., 2022 [50]	PTSD	Scuba diving with mindfulness exercises (the Bathysmed protocol)	6 days with 10 dives	↔ PCL-5 at post-test; ↓ Subscale intrusion symptoms (PCL-5) at post-test and 1-month follow-up; ↑ Mindfulness (FMI) at post-test; Large effect size Cohen’s *d* at 1-month follow-up; ↔ PCL-5 and FMI at 3-month follow-up.
6	Lang et al., 2019 [51]	PTSD	CBCT	10 weekly sessions (1 h for each)	↑ Social connectedness (SCS-R); ↓ PCL-5, PHQ-9, CAPS-5 (subscale hyperarousal), large effect size in hyperarousal, reexperiencing, negative alterations in cognitions. Medium effect size in empathy, mindful awareness, anxiety, rumination. Large effect size in depression. ↔ Differential Emotion Scale (DES): Positive and negative emotions; ↔ Alcohol consumption and all the other variables.
7	Yi. et al., 2022 [52]	PTSD from MVA	Kripalu yoga	6 sessions (45 min for each) for 12 weeks	↓ IES-R at post-test; ↓ Subscales intrusion and avoidance; ↔ Subscale hyperarousal; ↔ IES-R at 3-month follow-up; ↓ DASS-21 at post-test and 3-month follow-up and total score ˂ control; ↓ Subscales depression and anxiety; ↔ Subscale stress.
8	Somohano et al., 2022 [53]	PTSD-SUD	MBRP	8 sessions (1 h each) in a 4-week period	Higher duration (i.e., minutes per practice) of formal mindfulness practice → lower PTSD Symptoms (avoidance, arousal, reactivity, negative cognitions and mood in PCL-5) at 6-month follow-up; ↔ Informal practice did not predict any outcomes; ↔ Formal and informal practice did not predict reduction in intrusion symptoms (PCL-5) and craving at 6-month follow-up.
9	Müller-Engelmann et al., 2019 [54]	Interpersonal violence Childhood sexual or physical abuse Physical violence in adulthood	Trauma-adapted intervention from loving-kindness meditation and MBSR	8 individual sessions (1.30 h each)	↓ CAPS-5 at follow-up (especially on avoidance) (9 out of 12 did not meet PTSD criteria); ↓ DTS at post-test and follow-up; ↓ BDI-II at follow-up; ↓ Self-criticism at follow-up; ↔ BSI medium effect sizes; ↑ Mindfulness skills of nonjudging and acting with awareness; ↑ Attention to breath in MBE at follow-up; ↑ Self-compassion at follow-up; ↑ WHO-5 (75%): half of them at post-test.
10	Staples et al., 2022 [55]	PTSD	MBSG	10 weeks	↓ Hyperarousal and avoidance; ↓ PTSD symptoms; ↓ Anger and sleep disturbance; ↔ Depression, anxiety, post-traumatic growth, and health-related quality of life.
11	Aizik-Reebs et al., 2022 [56]	Traumatized and chronically stressed Forced displacement	MBTR-R	9 weekly sessions (2.5 h each)	↓ Post-test change in self-criticism (endorsement and drift rate); ↔ Post-test change in drift rates to self-compassion stimuli; ↑ Post-test increase in self-compassion (endorsement); Type of treatment (MBTR-R; Waitlist) → change of self-criticism (at post-test); → PTSD symptoms (HTQ) and depression (PHQ-9); Type of treatment (MBTR-R; Waitlist) → change of self-compassion (at post-test); → PTSD symptoms (HTQ), but not depression (PHQ-9),
12	Oren-Schwartz, 2023 [57]	Forced displacement	MBTR-R	9 weekly sessions (2.5 h each)	MBTR-R, relative to waitlist control → shame (no guilt) at post-test → PTSD symptom severity (HTQ subscale)/depression (PHQ-9) at post-test.
13	Kang, Sponheim & Lim, 2022 [58]	PTSD from combat	MBSR	8 weekly sessions	↓ PCL-5; ↑ Spontaneous alpha power (8–13 Hz) in the posterior electrode cluster but ↔ in the follow-up analysis; ↑ Task-related frontal theta power (4–7 Hz in 140–220 ms after stimulus); ↑ Frontal theta heartbeat-evoked brain responses (HEBR) (3–5 Hz and 265–336 ms after R peak); ↓ CAPS; ↓ PHQ; Type of treatment (MBSR, control) → frontal theta heartbeat evoked brain responses → PCL-5.
14	Fishbein et al., 2022 [59]	Cancer survivors	ACT	10 sessions	↓ Bull’s eye values BEVS (improvement); ↔ VLQ; ACT → SCS, EAC → IES-R; ACT → SCS, EAC, BEVS → CARS and general anxiety HADS-A (marginal mediation); ↑ SCS; ↑ EAC.
15	Mehling et al., 2018 [60]	PTSD	IE	36 sessions in 12 weeks (50 min each)	↓ CAPS-5 (average reduction of 31 points); ↑ FFMQ non-reactivity, pbserving; ↑ MAIA emotional awareness, self-regulation, body listening; ↑ PSOM total, focused attention, restful repose; PSOM and FFMQ non-reactivity → CAPS hyperarousal subscale/psychological WHOQOL (partial mediation).
16	Powers et al., 2022 [61]	PTSD and chronic trauma exposure to multiple events	Trauma-adapted MBCT group Combined interventions	8 weekly sessions (1.5 h each)	Good feasibility (75% completers) Good acceptability: high levels of satisfaction (CSQ-8) and several perceived benefits regarding physical state, emotional state, and interpersonal relationships; the most frequently reported barriers (PBPT): participation restrictions, stigma, lack of motivation, no availability of services, emotional concerns, misfit of therapy to needs, time constraints, and negative evaluation.
17	Killeen et al., 2023 [62]	PTSD; SUD	Trauma-adapted MBRP	8 weekly sessions	48 women met the definition of non-completers (attending < 75% sessions); ↓ Lowest rate of completion among unemployed women in the ICS control group, with low FFMQ; ↑ Higher rate of completion in women in TA-MBRP group with low PSS and high FFMQ; ↓ Both the TA-MBRP and ICS groups had low probability of completion for those with high PSS scores.
18	Somohano & Bowen, 2022 [63]	PTSD-SUD	Trauma-focused and gender-responsive MBRP	4 weekly sessions (1 h each)	↓ Craving (PACS) and PTSD symptoms (BSSS) in both conditions over the 12-month follow-up period and effect sizes similar to other PTSD-SUD interventions; ↑ Larger effect of craving and PTSD in both programs after 1 month; ↓ MBRP had lower BSSS at post-test and 1-month follow-up in comparison with TI-MBRP; TI-MBRP acceptability: homework practice was as expected (in both conditions); retention was below the target but 60%; attrition was higher (64%) at post-test and 1-month follow-up in Ti-MBRP than in MBRP. High satisfaction (OCSS).
19	Possemato et al., 2022 [64]	PTSD	PCBMT	4 weeks	↓ PTSD symptoms at post-test; ↓ Depression at 16–24 months follow-up; ↑ Health responsibility; ↑ Stress management, not feeling dominated by symptoms.
20	Classen et al., 2020 [65]	Childhood trauma, complex PTSD symptoms.	Trauma and the Body Group (TBG)	20-session program (h for each):	↑ Body awareness subscale (SBC); ↔ Body dissociation subscale (SBC); ↓ Anxiety (BAI); ↑ Soothing receptivity (SRS); ↔ PCL-5; SDQ-20; DES; PHLMS; IIP-32; ↓ BDI-II.
21	Gallegos et al., 2020 [66]	In	MBSR	8 weekly sessions	No statistical power to test between-group differences; Time effects in MBSR group: Improvement but ↔ divided and selective attention (UFOV) ↔ HRV by RMSSD but increase; ↓ DERS; ↓ PCL-5 at post-test and follow-up. Decrease for 50% of the total of participants.
22	Nguyen-Feng et al., 2020 [67]	PTSD and childhood interpersonal trauma histories	TCTSY	10 weekly sessions (1 h each)	TCTSY was most efficacious for those with fewer adult-onset interpersonal traumas. Within this subgroup, TCTSY was more effective in reducing PTSD than the active control condition. Clinician-rated PTSD, self-reported PTSD, and emotional control problems, although effects were relatively small-to-moderate. The efficacy of the intervention conditions was less predictable among those with a history of greater adult-onset interpersonal trauma.
23	Davis et al., 2020 [68]	PTSD	HYP; WLP	16 weekly sessions	↓ PCL-5; CAPS-5.
24	Fortuna et al., 2020 [69]	Dual diagnosis of SUD, depression, anxiety, and chronic stress	IIDEA; CBT + mindfulness Combined interventions	10 weekly sessions IIDEA trial (1 h each)	Intermediary variables: ↑WAI-SR (alliance), MAAS, and IMR (from a medium-to-small effect size) also at 6-month follow-up; Outcomes: ↓ Urine test and substance use ASI; PCL-10; GAD; PHQ-9; HSCL-20. Qualitative results: Participants found being listened without judgement, learning relaxation and emotional regulation techniques, gaining a sense of self-control, and managing the double diagnosis more useful.
25	Cox et al., 2019 [70]	Post discharge of critical illness	Mobile and telephone mindfulness program (awareness of breathing; body systems; emotion and mindful acceptance; and awareness of sound)	4 weekly sessions (1 h each)	Higher drop-out and less CSQ in mobile program. ↓ Similar decrease between mobile and telephone nindfulness in PHQ-9, GAD-7; PTSS at 3 months follow-up; ↓ Education program had a similar impact of mindfulness program on PTSS but less impact than others on PHQ-9 and GAD-7 at 3-months follow-up; ↔ CAMS-R and Brief-COPE.
26	Miller et al., 2021 [71]	PTSD	Mentoring + mindfulness program (Learning to Breathe, L2B)	4 mindfulness sessions (30 min each) in 12 weeks	↓ Child PTSD symptoms; ↓ Emotional impulsivity (DERS-SF); ↓ Difficult in engaging in goal-directed behavior (DERS-SF); ↔ Remaining variables.
27	Pradhan et al., 2018 [72]	Physical sexual and emotional abuse	TIMBER combined with a single sub-anesthetic dose of ketamine	12 weekly sessions (1 h each)	↑ Duration of response in TIMBER-K (compared to TIMBER-Placebo): On average, 34 days with no or minimal PTSD symptoms (PCL, CAPS) (twice longer than the remission with mindfulness therapy alone and 5-fold longer than Ket therapy alone); ↓ PCL and CAPS at relapse were lower than the pre-test; ↔ The average DSR (serine) plasma concentration was lower than basal DSR (but not significant); ↔ Positive correlation, but not significant, between DSR and PTSD severity.
28	Romaniuk et al., 2023 [73]	PTSD	CMT compared to CFT based on psychoeducational skills	12 biweekly group sessions (2 h each)	↓ Fear of compassion (FCS) towards others and self from pre-test to follow-up; ↓ Feelings of self-inadequacy (FSCRS) from pre-test to follow-up; ↓ Levels of external shame (OAS) at follow-up; ↓ PCL-5 in the ex-service personnel; ↓ Anxiety (DASS-21) at follow-up; ↓ Stress (DASS-21) at follow-up; ↔ PCL-5 in the partner group; ↔ Depression (DASS-21); ↑ Social safeness (SSPS) at follow-up; ↑ Quality of life and satisfaction (Q-LES-Q-SF) at post-test but not at follow-up; ↑ Relationship satisfaction (RAS) at post-test but not at follow-up.
29	Leach & Lorenzon, 2023 [74]	Traumatic experience of domestic violence	TM	9 individual and group sessions (1–2 h each): total 12 h in 8 weeks	↓ DASS-21 depression, anxiety, and stress severity scores; ↓ PCL-5 total symptom severity score; ↑ AQoL-8D utility score, superdomain scores, and domain scores (except for pain and senses domain scores). ADVERSE EFFECTS Twelve mild adverse events reported by six participants (i.e., nausea, headache, irritability, weight gain). Two participants self-reported a severe adverse event that they believed was related to the intervention (i.e., cold-sore, body feeling heavy).
30	Javidi et al., 2023 [75]	PTSD	Self-compassion therapy combined with CBT	12-session program of individualized CBT-based treatment	↑ SCS; ↓ K10; ↓ PHQ9; ↓ PCL-C; ↓ WSAS.
31	Bellehsen et al., 2022 [76]	PTSD	TM	16 sessions over 12 weeks (1 h each).	↓ PCL-5; BDI-II; BAI; ISI; ↓ 50% TM group reduced CAPS-5 and 50.0% no longer met the criteria for a PTSD diagnosis after 3 months; ↔ S-anger; ↔ Q-LES/Q-SF.
32	Gerdes et al., 2022 [77]	PTSD	LKM-S	1 session audio-taped (1.5 h)	↓ Self-reported hyperarousal state; ↓ SCL, meaning a reduction in sympathetic arousal; ↔ HRV response was not different from 0, meaning that the intervention may not have increased the parasympathetic activation (unlike what was expected); ↔ Social connectedness; State self-compassion at both pre and post time points were associated with PCL-5, trait self-compassion (SCS), and emotion suppression (ERQ); ↑ HR response (physiological arousal) (not, as expected, a decrease); ↑ HR response to directing compassion towards the self; ↑ Self-compassion state at the end of LKM.
33	Knabb et al., 2022 [78]	Exposure to crime-related events, physical and sexual experiences, and general disasters	Christian meditative intervention (Lectio Divina)	2 weeks	↓ PTSD symptoms; ↔ Positive effect (small effect size); ↔ Christian contentment; ↔ Christian gratitude; ↔ Anxiety, depression, stress (medium effect size).
34	Hodgdon et al., 2022 [79]	PTSD	IFS Combined interventions	16 individual sessions (1.5 h each)	↓ CAPS at post-test and 1-month follow-up; At 1-month follow-up, 92% of participants no longer met criteria for PTSD; ↓ DTS at post-test and 1-month follow-up; ↓ BDI at post-test and 1-month follow-up; ↓ SIDES total score at the 1-month follow-up; ↔ Somatization; ↔ SCS; ↑ Large effect size on trusting and medium effect sizes on attention regulation, self-regulation, and body listening; ↑ Not-sistracting subscale of MAIA just at 1-month follow-up; ↔ No significant time effect for other subscales of MAIA.
35	Tibbitts et al., 2021 [80]	Not revealed	TIY	From 2 to 10 sessions	↓ Reported decreased feeling pain or negative emotional states; ↑ Use of self-regulation skills was uniformly higher; ↑ Reported increased awareness of physical sensations (e.g., breathing and muscle movement); ↑ Students in the corrections and reentry sector had the largest benefit after beginning yoga. Adverse effects: For negative emotional states, only a few students reported feeling upset, anxious, or stressed after class. Fewer respondents from substance use treatment retrospectively reported feeling upset and anxious or stressed before yoga class. This group showed the least amount of change in self-regulation skills.
36	Kananian et al., 2020 [81]	Multiple trauma pre–post displacement	CA-CBT Combined interventions	12 sessions in 6 weeks (1.30 h each)	↓ PHQ-9; SSS-8; ↔ PCL-5; ↑ WHOQOL-BREF; ↑ ERS; ↑ GHQ-28 at both follow-up. At 1-year follow-up main effects were maintained
37	Schuurmans et al., 2021 [82]	PTSD	MUSE	6 weeks: 2 times a week for 15–20 min	↓ Basal activity of SNS (sympathetic nervous system); ↔ Reactivity of SNS and PNS (parasympathetic nervous system) to acute stress; ↑ HPA (hypothalamic–pituitary–adrenal axis) reactivity to acute stress.
38	Zalta et al., 2020 [83]	PTSD	ITP Combined interventions	3 weeks: 14 individual sessions of CPT, 13 sessions of group VPT, 13 session group mindfulness adapted from MBSR, and 12 sessions of yoga	↓ ISI in just 23.4%; ↓ PCL-5-18; PHQ-8; ↔ Baseline ISI did not predict PCL-5 and PHQ-8 across all time points, but larger improvements in ISI were associated with greater improvement in PCL-5 and PHQ.
39	Bandy et al., 2020 [84]	Several among natural disasters, severe accidents, sexual and criminal victimization, and combat experiences	TM	4 consecutive days (1.30 h daily) and weekly follow-up meetings and home practices	↓ PCL-C in experimental group after 15, 60, and 105 days of practice. In this point, PCL-C was not symptomatic anymore; ↓ BDI at both 60 and 105 days; ↓ BDI Depression and PTSD were highly correlated and decreased together through the practice. Regular TM practice predicted. ↓ PCL-C especially during the first 15 days of practice.
40	Zaccari et al., 2020 [85]	PTSD	Yoga protocol	10 weeks	↑ Life satisfaction; ↓ Depression, cortisol; ↔ Cognitive performance.
41	Ong et al., 2019 [86]	IPV	TSY	8 weekly sessions (1 h each)	↓ CAPS-5 (but one for floor effect): reduced number and severity. Enhanced physiological, intrapsychic functioning, emotional benefits, enhanced perceptions of self and others, shift in time perspective, interpersonal relationships, self-care, spiritual benefits, and positive coping strategies.
42	Reinhardt et al., 2018 [87]	PTSD	Kripalu yoga program	20 sessions in 10 weeks (1.30 h each)	CAPS-5 up to moderate PTSD symptoms at post-test in yoga group. ↔ Between differences in CAPS, PCL-M, and IES; ↓ Large effect PCL-M (correlated with PCL-C), self-reported PTSD symptoms were reduced in the yoga group (below the cutoff) while marginally increased in the control group, 51% drop out (higher in the yoga group). Self-selectors (from waitlist) improved more than randomized veterans in CAPS and PCL.

Note: ↑ = The variables improved and were statistically significant from baseline or from the control group; ↔ = no or no statistical significant difference from baseline or from the control group; ↓ = the variables declined and were statistically significant from baseline or from the control group; → = the independent variables significantly predict the outcome variables or the mediators.

## Data Availability

No new data were created or analyzed in this study. Data sharing is not applicable to this article.

## Supplementary Materials

The following supporting information can be downloaded at: https://www.mdpi.com/article/10.3390/healthcare13222825/s1, Table S1: Detailed Studies’ Characteristics.

## Abbreviations

**General Glossary**	
**AAOc**	Acceptance and Action Questionnaire-Cancer
**ANS**	Autonomous Nervous System
**AQoL-8D**	Australian Quality of Life (8-dimension)
**ASI**	Addiction Severity Index
**AUDIT-C**	Alcohol Use Disorders Identification Test
**BAI**	Beck Anxiety Inventory
**BDI**	Beck Depression Inventory
**BDI-II**	Beck Depression Inventory-II
**BEAQ**	Brief Experiential Avoidance Questionnaire;
**BEVS**	Bull’s-Eye Values Survey
**BPM**	Brief Problem Monitor
**BRIEF**	Behavioral Rating Inventory of Executive Function
**BSI**	Brief Symptom Inventory
**BSI-18**	Brief Symptom Inventory
**BSSS**	Brief Sensation Seeking Scale
**CAMS-R**	Cognitive and Affective Mindfulness Scale-Revised
**CAPS**	Clinician-Administered Post-traumatic Stress Disorder Scale
**CAPS-5**	Clinician-Administered PTSD Scale for DSM-5
**CARS**	Concerns About Recurrence Scale
**COPE**	Coping Orientation to the Problems Experienced
**CSQ**	Client Satisfaction Questionnaire
**CSQ-8**	Client Satisfaction Questionnaire
**CTQ-SF**	Childhood Trauma Questionnaire Short Form
**DASS-21**	Depression Anxiety and Stress Scale
**DERS**	Difficulties in Emotional Regulation Scale
**DES**	Dissociative Experiences Scale
**DES’**	Differential Emotions Scale
**DTS**	Davidson Trauma Scale
**EAC**	Emotional Approach Coping scale
**ECG**	Electrocardiogram
**ERQ**	Emotion Regulation Questionnaire
**ERS**	Emotion Regulation Scale
**FCS**	fears of compassion scales
**FFMQ**	Five-Facet Mindfulness Questionnaire
**FMI**	Freiburg Mindfulness Inventory
**FSCRS**	Forms of Self-Criticizing/attacking and Self-Reassuring Scale
**GAD-7**	Generalized Anxiety Disorder Scale
**GHQ-28**	General Health Questionnaire
**HADS-A**	Hospital Anxiety and Depression Scale-Anxiety subscale
**Ham-D**	Hamilton Depression Rating Scale
**HEBR**	Heartbeat-evoked brain response
**HR**	Heart Rate
**HRV**	Heart Rate Variability
**HSCL**	Hopkins Symptom Checklist
**HTQ**	The Harvard Trauma Questionnaire
**IASC**	Inventory of Altered Self-Capacities
**ICG**	Impedance Cardiography
**IES**	Impact of Events Scale
**IES-R**	Impact of Events Scale-Revised
**IIP-32**	Inventory of Interpersonal Problems
**IMR**	Illness Management and Recovery
**IPDE**	International Personality Disorder Examination
**IPVE**	Intimate Partner Violence exposure
**ISI**	Insomnia Severity Index
**K10**	Kessler Psychological Distress Scale
**LEC-5**	Life Events Checklist
**LKM**	loving-kindness meditation
**LKM-S**	loving-kindness meditation for self-compassion
**LSCL-R**	Life Stressor Checklist Revised
**LSI**	Leisure Score Index
**MAAS**	Mindfulness Awareness Attention Scale
**MAIA**	Multidimensional Assessment of Interoceptive Awareness
**MBE**	Mindful-Breathing Exercise
**MINI**	Mini International Neuropsychiatric Interview
**MoCA**	Montreal Cognitive Assessment
**NIH PROMIS**	National Institutes of Health’s Patient-Reported Outcomes Measurement Information System
**OAS**	Other as Shamer Scale
**OCSS**	Overall Course Satisfaction Survey
**OLBI**	Oldenburg Burnout Inventory
**PACS**	Penn Alcohol Craving Scale
**PANAS**	Positive and Negative Affect Schedule
**PBPT**	Perceived Barriers to Psychological Treatments
**PCL-5**	PTSD checklist for DSM-5
**PCL-C**	PTSD Checklist-Civilian Version
**PCL-M**	PTSD Checklist-Military Version
**PHLMS**	Philadelphia Mindfulness Scale
**PHQ**	Patient Health Questionnaire
**PHQ-8**	Patient Health Questionnaire Eight-item version
**PHQ-9**	Brief Patient Health Questionnaire for Depression
**PMLD**	Postmigration Living Difficulties Scale
**PROMIS**	Patient Reported Outcomes Measurement Information System 43-item version
**PSOM**	Positive States of Mind Scales
**PSQ**	Police Stress Questionnaire
**PSQI**	Pittsburgh Sleep Quality Index
**PSS**	PTSD Symptom Scale-Self Report
**PTSS**	The Post-Traumatic Stress Scale
**PWB**	Psychological Well-Being Scale
**Q-LES-SF**	Quality of Life Enjoyment and Satisfaction Questionnaire–Short Form
**RAS**	Relationship Assessment Scale
**RCT**	Randomized Controlled Trial
**REDS**	Reward-based Eating Drive Scale
**RMSSD**	Root Mean Square of Successive Differences
**RTSQ**	Ruminative Thought Style Questionnaire
**S-Ang**	State Anger scale
**SBC**	Scale of Body Connection
**SCID-I**	Structured Clinical Interview for DSM-IV
**SCL**	Skin Conductance Levels
**SCS**	Self-Compassion Scale
**SCS-R**	Social Connectedness Scale–Revised
**SCS-S**	Self-Compassion Scale-Short Form
**SDQ-20**	Somatoform Dissociation Questionnaire
**SIDES-SR**	Structured Interview for Disorders of Extreme Stress, Self-Report version
**SLESQ**	Stressful Life Events Screening Questionnaire
**SRET**	Self-Referential Encoding Task
**SRS**	Soothing Receptivity Scale
**SSGS**	State Shame and Guilt Scale
**SSPS**	Social Safeness and Pleasure Scale
**SSS-8**	Eight-item Somatic Symptom Scale
**SSST**	Sing-a-Song Stress Test
**STAXI-2**	State–Trait Anger Expression Inventory–2
**SUD**	Subjective Units of Distress Scale
**TEI**	Traumatic Events Inventory
**TEQ**	Toronto Empathy Questionnaire
**TF**	Time Frequency
**TLFB**	Timeline Follow Back
**TM**	Transcendental Meditation
**TRIER-C**	Trier Social Stress Task for Children
**TSK-11**	Tampa Scale of Kinesiophobia
**UFOV**	Useful Field of View Test
**VAS**	Visual Analogue scales
**VLQ**	Valued Living Questionnaire
**VU-AMS**	VU University Monitoring System
**WAI-SR**	Working Alliance Inventory-Short Revised
**WGO-5**	WHO-Five Well-Being Index
**WHOQOL**	World Health Organization Quality of Life, Brief Version
**WQL-8**	Work Limitations Questionnaire-Short Form
**WSAS**	Work and Social Adjustment Scale
**Table Glossary**	
**ACT**	Acceptance and Commitment Therapy
CA-CBT	Culturally adapted cognitive behavioral therapy
**CBCT**	Cognitively-based Compassion Training
**CBT**	Cognitive Behavioral Therapy
**CFT**	Compassion Focused Therapy
**CMT**	Compassioned Mind Training
**HYP**	Holistic Yoga Program
**IIDEA**	Intervention for dual problems and early action
**IE**	Integrative exercise
**IFS**	Internal Family System therapy
**IPV**	Intimate Partner Violence
**ITP**	Intensive treatment program
**LKM-S**	Listening to the compassion meditation
**MBCT**	Mindfulness-Based Cognitive Therapy
**MBRP**	Mindfulness-Based Relapse Prevention
**MBSG**	Mind-body skills group
**MBSR**	Mindfulness-Based Stress Reduction
**MBTR-R**	Mindfulness-Compassion Based Trauma Recovery
**MUSE**	Game-based meditation intervention
**MVA**	Motor vehicle accident
**PCBMT**	Primary Care Brief Mindfulness Training
**SUD**	Substance Use Disorder
**TCTSY**	Trauma Centered Trauma-Sensitive Yoga
**TIMBER**	Trauma Interventions using Mindfulness-Based Extinction and Reconsolidation
**TIY**	Trauma-informed Yoga
**TM**	Transcendental Meditation
**TSY**	Trauma-sensitive Yoga
**WLP**	Wellness Lifestyle Program
