# Supplementary material for: Do Contemplative Practices Promote Trauma Recovery? A Narrative Review from 2018 to 2023
Source: Healthcare (Basel). 2025 Nov 7;13(22):2825. doi: 10.3390/healthcare13222825 (PMC12652857; doi:10.3390/healthcare13222825)
Supplement: Supplementary file 1 [file healthcare-13-02825-s001.zip › healthcare-3782198-supplementary.pdf]

Table S1. Detailed Studies' Characteristics.

N	Authors	Study design	Sample size	Type of Trauma	Type of Contemplative practices	Duration	Measures	Main results
1	Jasbi et al., 2018 [21]	RCT with active control	48 male veterans; Mage=53 (SD=2,5)	PTSD	MBCT + Citalopram	8 weekly sessions (60-70 min each)	PCL-5; DASS	↓ PCL-5 (Re-experiencing the events, Avoidance, Negative mood and cognition, Hyperarousal) ↓ DASS (Depression, Anxiety, Stress)
2	Goldstein et al., 2018 [47]	RCT with waitlist and multiple assessment	47 (81% male); Mage= 46.8 (SD= 14.9); war veterans with PTSD, mostly and from racial or ethnic minority (60%).	PTSD	IE	36 sessions in 12 weeks (1 hr each)	SCID-I; CAPS-5; WHOQOL-BREF; Feasibility and Acceptability Questionnaire; Godin Leisure-Time Exercise Questionnaire.	↓CAPS-5 Tot. 31 point reduction at post-test ↓Subscale of hyperarousal ↑ LSI (more physical activity) ↑WHOQOL-BREF greater improvement in the psychological domain but a smaller improvement in the physical domain greater number of sessions attended was associated with an improvement in physical quality of life and psychological quality of life High levels of satisfaction
3	Chopin et al., 2020 [48]	RCT versus control	87 (61% male); Mage 51.41 (SD 11.32); 69 % African Americans; 49 completers (56,3%).	PTSD with comorbid chronic pain	Hatha Yoga	10 cohorts (2 to 8 weeks): 90 min each	PCL-5; PROMIS; Client Satisfaction Questionnaire-8; TSK-11.	↓ PTSD symptoms, kinesiophobia, depression and anxiety Follow-up results: ↔ Intrusion and avoidance symptoms ↑ Social role functioning PROMIS
4	Grupe et al., 2021[49]	RCT and follow-up	30 police officers;	Occupational stress	MBSR	8 weekly sessions	PSQ; OLBI with separate subscales for exhaustion and disengagement; PSQI; PCL-C); PROMIS; WLQ-8); PWB; PANAS; Creactive protein,	↓PSQ Operational stress and moderated by gender and years of police experience: younger men showed greater decline in stress also at follow-up;

			Mage= 38,4 (SD= 7,7).				diastolic/systolic blood pressure, resting pulse rate, skin conductance; Breath Count task.	↓ PCL at post-test and at 5-month follow-up;<br>↓ Exhaustion subscale of OLBI<br>↓PROMIS anxiety symptoms and depression symptoms;<br>↓PANAS Negative affect;<br>↔PROMIS subscales of pain interference; pain intensity, or physical functioning<br>↔ Disengagement subscale of OLBI<br>↔PANAS positive affect;<br>↔ Physical parameters<br>↑ Sleep quality PSQI<br>↑PWB;
5	Gibert et al., 2022 [50]	RCT with active control	34 people (Dive Group=17); (32,4% women);<br>Mage= 36 (SD= 6,9)	PTSD	Scuba diving with mindfulness exercises (the Bathysmed protocol)	6 days with 10 dives	PCL-5; FMI	↔PCL-5 at post-test but:<br>↓Subscale Intrusion symptoms (PCL-5) at post-test and 1-month follow-up;<br>↑ Mindfulness (FMI) at Post-test;<br>Large effect size Cohen's *d* at 1-month follow-up;<br>↔ PCL-5 and FMI at 3-month follow-up
6	Lang et al., 2019 [51]	Pilot RCT	28 Veterans;<br>Mage=49.6 (SD=16.2)	PTSD	CBCT	10 weekly sessions (1 hr for each)	CAPS-5; PCL-5; PHQ-9; BSI-18; STAXI-2;<br>Sleep-Related Disturbance Measures from NIH PROMIS; AUDIT-C; SCS-R; SCS; PHLMS; TEQ; RTSQ; DES'	↑ Social connectedness (SCS-R)<br>↓PCL-5, PHQ-9, CAPS-5 (subscale Hyperarousal), large effect size in hyperarousal, reexperiencing, negative alterations in cognitions<br>Medium effect size in empathy, mindful awareness, anxiety, rumination<br>Large effect size in depression

								↔ Differential Emotion Scale (DES): Positive and Negative Emotions<br>↔Alcool Consumption and all the other variables
7	Yi. et al., 2022 [52]	RCT with active control	94 women;<br>Mage= 40.8 (SD= 13,2), Most drivers with almost two months since MVA	PTSD from MVA	Kripalu Yoga	6 sessions (45 min for each) for 12 weeks	IES-R; DASS-21	↓IES-R at post-test<br>↓Subscales Intrusion and Avoidance<br>↔Subscale Hyperarousal<br>↔IES-R at 3-month follow-up<br>↓DASS-21 at post-test and 3-month follow-up and Tot score<Control<br>↓Subscales Depression and Anxiety<br>↔Subscale Stress
8	Somohano et al., 2022 [53]	Pilot RCT and follow-up	23 women;<br>Mage= 36.1 (SD= 7.9)	PTSD-SUD	MBRP	8 sessions (1 hr each) in a 4-week period	PCL-5; PACS; self-reported minutes of daily meditation practice, and self-reported frequency of daily engagement in mindfulness skills to everyday activities	Higher duration (i.e., minutes per practice) of formal mindfulness practice → lower PTSD Symptoms (avoidance, arousal, reactivity, negative cognitions and mood in PCL-5) at 6-month follow-up;<br>↔Informal practice did not predict any outcomes.<br>↔Formal and informal practice did not predict reduction in intrusion symptoms (PCL-5) and craving at 6-month follow-up.
9	Muller-Engelmann et al., 2019 [54]	RCT with multiple weekly assessments and follow-up	14 (78,6% women);<br>Mage=41.14 (SD= 12.30); PTSD patients	Interpersonal violence<br>Childhood sexual or physical abuse;	Trauma-adapted intervention from loving-kindness meditation and MBSR	8 individual sessions (1.30 hr each)	CAPS-5; SCID-I; IPDE; LEC-5; DTS; BSI; BDI-II; WHO-5; FFMQ; SCS; MBE.	↓CAPS-5 at follow-up (especially on avoidance) (9 out of 12 did not meet PTSD criteria);<br>↓DTS at post-test and follow-up;

				Physical violence in adulthood				↓BDI-II at follow-up;<br>↓ self-criticism at follow-up<br>↔BSI medium effect sizes<br>↑mindfulness skills of nonjudging and acting with awareness<br>↑ attention to breath in MBE at follow-up<br>↑self-compassion at follow-up<br>↑WHO-5 (75%): half of them at post-test
10	Staples et al., 2022 [55]	RCT with active control and follow-up	108 veterans (96% male);<br>Mage 55.97 (SD=11,7).	PTSD	MBSG	10 weeks	PCL- M; STAXI-2; PSQI; PHQ-9.	↓ Hyperarousal and avoidance<br>↓ PTSD symptoms<br>↓ Anger and sleep disturbance<br>↔ Depression, anxiety, post traumatic growth and health related quality of life
11	Aizik-Reebs et al., 2022 [56]	RCT with waitlist and follow-up	158 Eritrean asylum-seekers (46% female);<br>Mage=31.8 (SD=5.2);	Traumatized and chronically stressed;<br>Forced displacement	MBTR-R	9-weekly sessions (2.5 hr each)	HTQ; PHQ-9; SRET	↓Posttest change in self-criticism (endorsement and drift rate)<br>↔Posttest change in drift rates to self-compassion stimuli<br>↑ Posttest increase in self-compassion (endorsement)<br>Type of treatment (MBTR-R; Waitlist) →change of self-criticism (at post-test)<br>→ PTSD symptoms (HTQ) and depression (PHQ-9);<br>Type of treatment (MBTR-R; Waitlist) →change of self-compassion (at post-test)<br>→ PTSD symptoms (HTQ), but not depression (PHQ-9)

12	Oren-Schwarz, 2023 [57]	RCT with active control	158 Eritrean asylum-seekers (55.7% female); Mage= 31,8 (SD= 5.2)	Forced displacement	MBTR-R	9-weekly sessions (2.5 hr each)	HTQ; PHQ-9; SSGS; PMLD	MBTR-R, relative to waitlist control →shame (no guilt) at post-test → PTSD symptom severity (HTQ subscale) / Depression (PHQ-9) at post-test
13	Kang, Sponheim & Lim, 2022 [58]	RCT with active control (Present-centered group therapy)	98 veterans with PTSD symptoms (PCL) (14,3% women); Mage= 58.6 (SD= 10.4)	PTSD from combat	MBSR	8 weekly sessions	PCL-5; CAPS; PHQ-9; PHQ-15<br>EEG recorded with BioSemi Active Two EEG system in resting-meditation-resting procedure<br>ECG<br>Flanker cognitive task (as attentional task)<br>Spectral power of theta and alpha frequency oscillations of the spontaneous EEG and the TF;<br>HEBR	↓ PCL-5<br>↑ spontaneous alpha power (8–13 Hz) in the posterior electrode cluster but ↔ in the follow-up analysis<br>↑task-related frontal theta power (4–7 Hz in 140–220 ms after stimulus)<br>↑ frontal theta heartbeat-evoked brain responses (HEBR) (3–5 Hz and 265–336 ms after R peak).<br>↓ CAPS<br>↓ PHQ<br>Type of treatment (MBSR, Control)→ frontal theta heartbeat evoked brain responses→ PCL-5
14	Fishbein et al., 2022 [59]	RCT with active control	134 (88% female); Mage= 56.24 (SD=11.58).	Cancer survivors	ACT	10 sessions	Mediators: SCS; BEAQ; BEVS; VLQ; EAC.<br>Outcomes: HADS; IES-R; CARS.	↓ Bull's eye Values BEVS (improvement)<br>↔VLQ<br>ACT →SCS, EAC→IES-R<br>ACT→SCS, EAC, BEVS→ CARS and General Anxiety HADS-A (marginal mediation)<br>↑SCS

								↑ EAC
15	Mehling et al., 2018 [60]	RCT with waitlist and multiple assessment	47 (81% male);<br>Mage =46.8; from 24 to 69<br>war veterans with PTSD and from racial or ethnic minority (60%).	PTSD	IE	36 sessions in 12 weeks (50 min each)	FFMQ; MAIA; PSOM; CAPS-5; WHOQOL.	↓CAPS-5 (average reduction of 31 points)<br>↑FFMQ Non reactivity, Observing<br>↑ MAIA Emotional Awareness, Self-Regulation, Body Listening;<br>↑PSOM total, Focused Attention, Restful Repose<br>PSOM and FFMQ Non-Reactivity → CAPS Hyperarousal subscale/ Psychological WHOQOL (partial mediation)
16	Powers et al., 2022 [61]	RCT with active control and follow-up	42 Black adults (85% women);<br>Age from 18 to 65;	PTSD and chronic	Trauma adapted MBCT group<br>Combined interventions	8-weekly sessions (1,5 hr each)	TEI; PHQ-9; CAPS-5; MINI for screening; CSQ-8; PBPT	Good feasibility (75% completers)<br>Good Acceptability: high levels of satisfaction (CSQ-8) and several perceived benefits regarding physical, emotional state and interpersonal relationships; The most frequently reported barriers (PBPT): participation restrictions, stigma, lack of motivation, no availability of services, emotional concerns, misfit of therapy to needs, time constraints, and negative evaluation.
			(55.5% of MBCT, 87.5% of WLC)	trauma exposure to multiple events				
17	Killeen et al., 2023 [62]	RCT with active control Integrative Coping Skills (ICS)	90 Women (53,3% non-completers);<br>Age from 18 to 65	PTSD; SUD	Trauma adapted MBRP	8 weekly sessions	Rate of Retention in the treatment; PSS; FFMQ; MINI; CAPS-5; TLFB; DERS	48 women met the definition of non-completers (attending < 75% sessions);<br>↓ Lowest rate of completion among unemployed women in the ICS control group, with low FFMQ;

							↑ Higher rate of completion in women in TA-MBRP group with low PSS and high FFMQ;<br>↓ Both the TA-MBRP and ICS groups had low probability of completion for those with high-PSS scores.
18	Somohano & Bowen, 2022 [63]	Single cluster-randomized repeated measures design	83 women (30% ethnic minority);<br>Mage= 36.1 (SD= 7.9)	PTSD-SUD	Trauma-focused and gender responsive<br>MBRP	4 weekly sessions (1 hr each)	For PTSD symptom severity in primary care settings: BSSS; PACS;<br>Acceptability: homework compliance and<br>course satisfaction; self-reported duration of formal practices and frequency of informal practices<br>Feasibility: recruitment, enrolment, and retention.<br>Satisfaction: OCSS	↓Craving (PACS) and PTSD symptoms (BSSS) in both conditions over the 12-month follow-up period and effect sizes similar to other PTSD-SUD interventions<br>↑Larger effect of Craving and PTSD in both programs after 1-month<br>↓MBRP had lower BSSS at post-test and 1-month follow-up in comparison with TI-MBRP;<br>TI-MBRP acceptability: homework practice was as expected (in both conditions), retention was below the target but 60%; attrition was higher (64%) at post-test and 1-month follow-up in Ti-MBRP than in MBRP. High satisfaction (OCSS).
19	Possemato et al., 2022 [64]	RCT with active control and follow-up	55 primary care veterans	PTSD	PCBMT	4 weeks	PCL-5; PHQ-9; Health responsibility; Stress management; Not feeling dominated by symptoms	↓ PTSD symptoms at post-test<br>↓Depression at 16-24 months follow-up<br>↑Health responsibility<br>↑Stress Management, not feeling dominated by symptoms.
20	Classen et al., 2020 [65]	RCT with waitlist and follow-up	32 women;<br>Mage =43.5(SD= 10)<br>Eligible if they had previous group experience	Childhood trauma, complex PTSD symptoms.	Trauma and the Body Group (TBG)	20-session program (hr for each):	CTQ-SF; LSCL-R; SBC; BAI; SRS; PCL-5; SDQ-20); DES; BDI-II; PHLMS; IIP-32	↑Body awareness subscale (SBC)<br>↔ Body dissociation subscale (SBC)<br>↓ Anxiety (BAI)<br>↑Soothing receptivity (SRS)

21	Gallegos et al., 2020 [66]	RCT with active control and follow-up	29 women (65% in MBSR group);<br>Mage 42.7 (SD=13.1);<br>50% Black	In	MBSR	8 weekly sessions	IPVE; LEC-5; PCL-5; DERS; UFOV; RMSSD between normal heartbeats during a 5-min rest period and a sub-sequent trauma imagery task	↔ PCL-5; SDQ-20; DES; PHLMS; IIP-32<br>↓ BDI-II<br>No statistical power to test between-group differences;<br>Time effects in MBSR group: Improvement but ↔ divided and selective attention (UFOV)<br>↔ HRV by RMSSD but increase<br>↓DERS<br>↓PCL-5 at post-test and follow-up. Decrease for 50% of the total of participants.
22	Nguyen-Feng et al., 2020 [67]	RCT	64 women	PTSD and childhood interpersonal trauma histories	TCTSY	10 weekly sessions (1hr each)	SLESQ; CAPS; DTS; BDI-II; DES; IASC	TCTSY was most efficacious for those with fewer adult-onset interpersonal traumas. Within this subgroup, TCTSY was more effective in reducing PTSD than the active control condition. clinician-rated PTSD, selfreported PTSD, and emotional control problems, although effects were relatively small to moderate.<br>The efficacy of the intervention conditions was less predictable among those with a history of greater adult-onset interpersonal trauma.
23	Davis et al., 2020 [68]	RCT and follow-up	209 participants (91.4% veterans; 66% male; 61.7% White)<br>Mage= 50.6 (SD= 12.9)	PTSD	HYP; WLP	16 weekly sessions	CAPS-5; PCL-5;	↓PCL-5; CAPS-5
24	Fortuna et al., 2020 [69]	RCT	341 Ispanic migrants (24% US and 76% Spain) (172 assigned to IIDEA);	Dual diagnosis of SUD,	IIDEA; CBT + Mindfulness.	10 weekly sessions IIDEA trial (1 hr each)	Qualitative report on what was useful of the intervention (content analysis); WAI-SR;	Intermediary variables: ↑WAI-SR (alliance), MAAS and IMR

		with active control and follow-up	Age from 18 to 70	depression, anxiety, and chronic stress	Combined<br><br>interventions		IMR; MAAS; ASI; Lite and urine test results; HSCL-20; GAD-7; PCL-10; PHQ-9	(from a medium to small effect size) also at 6-month follow-up;<br><br>Outcomes: ↓ Urine test and substance use ASI; PCL-10; GAD; PHQ-9; HSCL-20<br><br>Qualitative results: participants found more useful being listened without judgement, learning relaxation and emotional regulation techniques, gaining a sense of self-control, and managing the double diagnosis.
25	Cox et al., 2019 [70]	Pilot RCT and follow-up	80 allocated to mobile/telephone mindfulness (77,5%)<br><br>or education (22,5%); 56,2% men;<br><br>Mage= 49.5;<br><br>admitted for a surgical or trauma diagnosis (69%)	Post discharge of critical illness	Mobile and telephone mindfulness program<br><br>(awareness of breathing;<br><br>body systems; emotion<br><br>and mindful acceptance,<br><br>and awareness of sound)	4 weekly sessions (1 hr each)	System Usability Scale; CSQ; Rate of completion of the program; PTSS; GAD-7; PHQ-9; CAMS-R; Brief COPE; qualitative data: Report and rate the severity of perceived stressors, open-ended feedback on the app group	Higher drop-out and less CSQ in Mobile program<br><br>↓Similar decrease between Mobile and Telephone Mindfulness in PHQ-9, GAD-7; PTSS at 3 months follow-up;<br><br>↓Education program had a similar impact of Mindfulness Program on PTSS but less impact than others on PHQ-9 and GAD-7 at 3-months follow-up;<br><br>↔CAMS-R and Brief-COPE
26	Miller et al., 2019 [71]	RCT with active control and follow-up	35 adolescents;<br><br>Mage= 12.9 (SD= 1,8);<br><br>(37% female)<br><br>32 parents;<br><br>Mage= 44.7 (SD= 9,8);	PTSD	Mentoring + mindfulness program (Learning to Breathe, L2B)	4 mindfulness sessions (30 min each) in 12 weeks	Internalizing subscale of BPM; Child PTSD Symptom Scale on distressing events; PROMIS Sleep-Related Disturbance—SF, PROMIS Pediatric Physical Activity-SF; (REDS); (BRIEF), DERS-SF; FFMQ-SF	↓ Child PTSD symptoms<br><br>↓Emotional impulsivity (DERS-SF)<br><br>↓Difficult in engaging in goal-directed behavior (DERS-SF)<br><br>↔Remaining variables

			(80% female)					
27	Pradhan et al., 2018 [72]	Pilot RCT with active control and follow-up	20 subjects (60% female)<br><br>Age 30-60	Physical sexual and emotional abuse	TIMBER combined with a single sub-anesthetic dose of ketamine.	12 weekly sessions (1 hr each)	PCL; CAPS; Ham-D; BAI; MoCA	↑Duration of response in TIMBER-K (compared to TIMBER-Placebo): in average 34 days with no or minimal PTSD symptoms (PCL, CAPS) (twice longer than the remission with Mindfulness Therapy alone and 5-fold longer than Ket therapy alone);<br><br>↓PCL and CAPS at the relapse were lower than the pre-test;<br><br>↔the average DSR (Serine) Plasma Concentration was lower than basal DSR (but not significant)<br><br>↔Positive correlation but not significant between DSR and PTSD severity
28	Romaniuk et al., 2023 [73]	Uncontrolled Clinical Trial and follow-up	12 couples (50% male);<br><br>Mage= 61.90 (SD= 11.09) for the males Mage=61.33 (SD= 8.65) for the females	PTSD	CMT compared to CFT based on psychoeducational skills.	12 biweekly group sessions (2 hr each)	FCS, FSCRS, OAS, SSPS, Depressive experiences questionnaire—Self-criticism subscale, PCL-5, DASS-21, Q-LES-Q-SF, RAS, Post-program feedback questionnaire.	↓ fear of compassion (FCS) towards others and self from pre-test to follow-up<br><br>↓ feelings of self-inadequacy (FSCRS) from pre-test to follow-up<br><br>↓ levels of external shame (OAS) at follow-up<br><br>↓ PCL-5 in the ex-service personnel<br><br>↓ Anxiety (DASS-21) at follow-up.<br><br>↓ Stress (DASS-21) at follow-up.<br><br>↔ PCL-5 in the partner group<br><br>↔ Depression (DASS-21)<br><br>↑ social safeness (SSPS) at follow-up<br><br>↑ Quality of life and satisfaction (Q-LES-Q-SF) at post-test but not at follow-up<br><br>↑ Relationship satisfaction (RAS) at post-test but not at follow-up

29	Leach & Lorenzon, 2023 [74]	RCT with active control and follow-up	42 women;<br><br>Mage 47.8 (SD= 12.3);<br>Most unemployed/retired (57.1%) and had experienced domestic violence more than 12 months ago (64.3%).	Traumatic experience of domestic violence	TM	9 individual and group sessions (1–2 hr each): tot 12 h in 8 weeks	AQoL8D, DASS-21, PCL-5, subjective experience was assessed through open-ended questions in the data collection form and trial exit form.	↓ DASS-21 depression, anxiety and stress severity scores<br>↓ PCL-5 Total Symptom Severity Score<br><br>↑ AQoL-8D utility score, superdomain scores and domain scores (except for pain and senses domain scores)<br><br>ADVERSE EFFECTS<br><br>Twelve mild adverse events reported by six participants (i.e. nausea, headache, irritability, weight gain). Two participants self-reported a severe adverse event that they believed was related to the intervention (i.e. cold-sore, body feeling heavy).
30	Javidi et al., 2023 [75]	RCT with active control	82 participants with a diagnosis of depressive disorder or PTSD;<br>Mage= 40.3 years (SD= 12.0)	PTSD	Self-Compassion Therapy combined with CBT	12 session program of individualised CBT-based treatment f	SCS, K10, PHQ9; PCL-C; WSAS.	↑ SCS<br><br>↓ K10<br>↓ PHQ9<br>↓ PCL-C<br>↓ WSAS
31	Bellehsen et al., 2022 [76]	RCT with active control and follow-up	40 veterans (85% male);<br><br>Mage=51.6 (SD= 11.4);<br><br>Non caucasian (42.5%).	PTSD	TM	16 sessions over 12 weeks (1 hr each).	CAPS-5; PCL-5; BDI-II; BAI; S-Anger subscale of STAXI-2; Q-LES-Q-SF; ISI.	↓PCL-5; BDI-II; BAI; ISI;<br><br>↓50% TM group reduced CAPS-5 and 50.0% no longer met the criteria for a PTSD diagnosis after 3 months<br>↔S-anger<br>↔Q-LES/ Q-SF
32	Gerdes et al., 2022 [77]	Uncontrolled Clinical Trial	56 veterans (92.9% male);<br><br>Mage= 52.1 (SD= 12.9).	PTSD	LKM-S	1 session audio-taped (1.5 hr)	HR and HRV from ECG;<br>SCL; ERQ; PCL-5; PHQ-9; VAS.	↓ self-reported hyperarousal state<br>↓ SCL, meaning a reduction in sympathetic arousal

								↔HRV response was not different from 0, meaning that the intervention may not have increased the parasympathetic activation (unlike what was expected)<br>↔ Social Connectedness<br>State self-compassion at both pre and post time points were associated with PCL-5, trait self-compassion (SCS) and emotion suppression (ERQ)<br>↑HR response (physiological arousal) (not as expected a decrease)<br>↑HR response to directing compassion towards the self<br>↑ self-compassion state at the end of LKM
33	Knabb et al., 2022 [78]	RCT with active control	26 participants belonging to Christian religion; Mage= 42,8 (SD=12,80);	Exposure to crime related events, physical and sexual experiences and general disasters	Christian Meditative Intervention (Lectio Divina)	2 weeks	Christian Contentment Scale; Christian Gratitude Scale; Positive and Negative Affect Schedule; Trauma Symptom Checlist-40	↓ PTSD symptoms<br>↔ Positive affect (small effect size)<br>↔ Christian contentment<br>↔ Christian gratitude<br>↔ Anxiety, depression, stress (medium effect size)
34	Hodgdon et al., 2022 [79]	Uncontrolled Pilot Clinical Trial and follow-up	17 participants (76% women);<br>Mage=46; range 28-58<br>76% exposed to two types of trauma prior to the age of 18; and current diagnosis of PTSD on the CAPS and clinical depression.	PTSD	IFS<br>Combined<br>interventions	16 individual sessions (1,5 hr each)	DTS; CAPS; BDI; SIDES-SR; SCS; MAIA.	↓CAPS at post-test and 1-month follow-up<br>At 1-month follow up, 92% of participants no longer met criteria for PTSD<br>↓DTS at post-test and 1-month follow-up<br>↓ BDI at post-test and 1-month follow-up

								↓SIDES total score at the 1-month follow up<br>↔Somatization<br>↔SCS<br>↑ Large effect size on Trusting and medium effect sizes on Attention Regulation, Self-Regulation and Body Listening<br>↑Not-Distracting subscale of MAIA just at 1-month follow-up<br>↔ No significant time effect for other subscales of MAIA
35	Tibbitts et al., 2021 [80]	Retrospective pre-post approach	152 students (59% woman), community of colour (44 %);<br>73% ≥ 21 years;<br>51% attended trauma-informed yoga classes in the corrections and reentry sector, 21% in the substance use treatment and recovery, 28% in community and mental health sectors.	Not revealed	TIY	From 2 to 10 sessions	Survey instrument: Not standardized questionnaire developed ad hoc informed by the framework of self-regulation to evaluate: self-regulation, perceived emotional and physical wellbeing.	↓Reported decreased feeling pain or negative emotional states<br>↑Use of self-regulation skills was uniformly higher.<br>↑Reported increased awareness of physical sensations (e.g. breathing and muscle movement)<br>↑Students in the corrections and reentry sector had the largest benefit after beginning yoga.<br>Adverse effects: For negative emotional states, only few students reported feeling upset, anxious, or stressed after class.<br>Fewer respondents from substance use treatment retrospectively reported feeling upset and anxious or stressed before yoga class. This group showed the least amount of change in self-regulation skills

36	Kananian et al., 2020 [81]	RCT with waitlist and follow-up	24 male; Mage= 22.1 (SD=3.6); refugees diagnosed with *DSM-5* PTSD, major depressive disorder, and anxiety disorder; with elementary school degree.	Multiple trauma pre-post displacement	CA-CBT Combined interventions	12 sessions in 6 weeks (1.30 hr each)	GHQ-28; PCL-5; PHQ-9; SSS-8; WHOQOL-BREF; ERS.	↓ PHQ-9; SSS-8; ↔PCL-5 ↑WHOQOL-BREF ↑ERS ↑GHQ-28 at both follow-up; At 1-year follow-up main effects were maintained
37	Schuurmans et al., 2021 [82]	RCT versus TAU	77 adolescents (59.7% male); Mage= 15.25 (SD = 1.79).	PTSD	MUSE	6 weeks: 2 times a week for 15-20 minutes	Basal ANS activity using ECG and ICG by VU-AMS. Basal HPA axis activity using hC levels pg/mg hair. TRIER-C combined with SSST.	↓ Basal activity of SNS (Sympathetic Nervous System) ↔ Reactivity of SNS and PNS (Parasympathetic Nervous System) to acute stress ↑ HPA (Hypothalamic-Pituitary-Adrenal Axis) reactivity to acute stress
38	Zalta et al., 2020 [83]	RCT with 10 assessments	165 veterans (64,2% male); Mage= 40.8 (SD= 9.57); most white; most who served After 9/11; most retired; with PTSD.	PTSD	ITP Combined interventions	3-week: 14 individual sessions of CPT, 13 sessions of group VPT, 13 session group mindfulness adapted from MBSR and 12 sessions of yoga	ISI; PCL-5-18; PHQ-8.	↓ ISI in just 23.4%; ↓ PCL-5-18; PHQ-8 ↔ baseline ISI did not predict PCL-5 and PHQ-8 across all time points but larger improvements in ISI was associated with greater improvement in PCL-5 and PHQ
39	Bandy et al., 2020 [84]	Uncontrolled Pilot Clinical Study	116 (61,2% women); Mage= 20.6 (SD= 2.75);	Several among natural disasters, severe accidents,	TM	4 consecutive days (1.30 hr daily), and weekly follow-up meetings and home practices	PCL-C; Trauma history Questionnaire; BDI.	↓PCL-C in experimental group after 15, 60 and 105 days of practice. In this point, PCL-C was not symptomatic anymore. ↓BDI at both 60 and 105 days

			South African students in experimental group with PTSD (PCL-C >44); 61 (70,5% women) in the waiting list: 34 participants also met DSM-IV criteria in a clinician's diagnosis.	sexual and criminal victimization and combat experiences				↓ BDI Depression and PTSD were highly correlated and decreased together through the practice; Regular TM practice predicted ↓PCL-C especially during the first 15 days of practice
40	Zaccari et al., 2020 [85]	Uncontrolled Clinical Trial	27 veterans; final sample (N = 17), 41% endorsed military sexual trauma (85% of women and 10% of men).	PTSD	Yoga protocol	10 weeks	Response Inhibition; PTSD symptoms; depression; sleep disorder; quality of life; neurocognitive complaints. Cognitive functioning, self-report measures of mental health symptoms, and salivary cortisol were measured within two weeks prior to beginning and following completion.	↑ Life satisfaction ↓ depression, cortisol ↔ cognitive performance
41	Ong et al., 2019 [86]	Collective case study design	3 women; Age from 26 to 52; PTSD who have left the abusive relationship for at least 6 months.	IPV	TSY	8 weekly sessions (1 hr each)	CAPS-5; SUD; pre- and post-TSY scaling question worksheet observations on how they made meaning of their recovery; semistructured interviews.	↓CAPS-5 (but one for floor effect): reduced number and severity Enhanced physiological, intrapsychic functioning, emotional benefits, enhanced perceptions of self and others, shift in time perspective, interpersonal relationships, self-care, spiritual benefits and positive coping strategies.
42	Mehling et al., 2018 [60]	RCT with waitlist and multiple assessment	47 (81% male); Mage =46.8; from 24 to 69	PTSD	IE	36 sessions in 12 weeks (50 min each)	FFMQ; MAIA; PSOM; CAPS-5; WHOQOL.	↓CAPS-5 (average reduction of 31 points) ↑FFMQ Non reactivity, Observing

42	Reinhardt et al., 2018 [87]	RCT with waitlist	51 (11.8 % female);<br><br>Mage= 47.76 (SD= 13.77);<br><br>(out of 74 participants screened).	PTSD	Kripalu Yoga Program	20 sessions in 10 weeks (1.30 hr each)	PTSD Checklist (PCL-C and M); CAPS-5; IES-R.	CAPS-5 up to moderate PTSD symptoms at post-test in Yoga group but<br><br>↔ between differences in CAPS, PCL-M and IES<br><br>↓ large effect PCL-M (correlated with PCL-C), self-reported PTSD symptoms were reduced in the yoga group (below the cutoff) while marginally increased in the control group, 51% drop out (higher in the Yoga Group),<br><br>Self-selectors (from Waitlist) improved more than Randomized Veterans in CAPS and PCL.

Note:
↑= the variables improved and were statistically significant from baseline or from the control group;
↔= no or no statistically significant difference from baseline or from the control group;
↓=the variables declined and were statistically significant from baseline or from the control group;
→=the independent variables significantly predict the outcome variables or the mediators.
